# High-resolution mapping reveals that microniches in the gastric glands control *Helicobacter pylori* colonization of the stomach

**DOI:** 10.1371/journal.pbio.3000231

**Published:** 2019-05-02

**Authors:** Connie Fung, Shumin Tan, Mifuyu Nakajima, Emma C. Skoog, Luis Fernando Camarillo-Guerrero, Jessica A. Klein, Trevor D. Lawley, Jay V. Solnick, Tadashi Fukami, Manuel R. Amieva

**Affiliations:** 1 Department of Microbiology and Immunology, Stanford University School of Medicine, Stanford, California, United States of America; 2 Department of Molecular Biology and Microbiology, Tufts University School of Medicine, Boston, Massachusetts, United States of America; 3 Department of Biology, Stanford University, Stanford, California, United States of America; 4 Center for Comparative Medicine, University of California, Davis School of Medicine, Davis, California, United States of America; 5 Host-Microbiota Interactions Laboratory, Wellcome Sanger Institute, Wellcome Genome Campus, Hinxton, United Kingdom; 6 Department of Pediatrics, Stanford University School of Medicine, Stanford, California, United States of America; 7 Department of Medicine, University of California, Davis School of Medicine, Davis, California, United States of America; 8 Department of Microbiology and Immunology, University of California, Davis School of Medicine, Davis, California, United States of America; New York University School of Medicine, UNITED STATES

## Abstract

Lifelong infection of the gastric mucosa by *Helicobacter pylori* can lead to peptic ulcers and gastric cancer. However, how the bacteria maintain chronic colonization in the face of constant mucus and epithelial cell turnover in the stomach is unclear. Here, we present a new model of how *H*. *pylori* establish and persist in stomach, which involves the colonization of a specialized microenvironment, or microniche, deep in the gastric glands. Using quantitative three-dimensional (3D) confocal microscopy and passive CLARITY technique (PACT), which renders tissues optically transparent, we analyzed intact stomachs from mice infected with a mixture of isogenic, fluorescent *H*. *pylori* strains with unprecedented spatial resolution. We discovered that a small number of bacterial founders initially establish colonies deep in the gastric glands and then expand to colonize adjacent glands, forming clonal population islands that persist over time. Gland-associated populations do not intermix with free-swimming bacteria in the surface mucus, and they compete for space and prevent newcomers from establishing in the stomach. Furthermore, bacterial mutants deficient in gland colonization are outcompeted by wild-type (WT) bacteria. Finally, we found that host factors such as the age at infection and T-cell responses control bacterial density within the glands. Collectively, our results demonstrate that microniches in the gastric glands house a persistent *H*. *pylori* reservoir, which we propose replenishes the more transient bacterial populations in the superficial mucosa.

## Abstract 2

High-resolution imaging of bacterial populations in whole infected organs reveals a novel mechanism of mucosal colonization, where the occupation of a specialized microenvironment, or "microniche," in the gastric glands allows Helicobacter pylori to establish a stable bacterial reservoir in the stomach that enhances bacterial persistence and colonization resistance.

## Introduction

All epithelial surfaces in the human body are colonized by bacteria. These mucosal communities may either confer a healthy microbiota or initiate chronic inflammatory diseases and recurrent infections. Understanding how bacteria establish themselves, survive mucosal clearance mechanisms, and compete for limited resources to promote chronic colonization is of critical importance—both for enhancing colonization of beneficial microbes and for eradicating pathogens that cause chronic infection.

Half of the world’s population is colonized with *Helicobacter pylori*, a highly specialized bacterium that lives only in the human stomach [1]. Infection is typically acquired in childhood and persists for life [2]. Although most individuals are colonized asymptomatically, chronic *H*. *pylori* infection is the strongest risk factor for developing peptic ulcers and gastric cancer [3]. To survive in the harsh environment of the stomach, *H*. *pylori* avoid the acidic lumen by colonizing a narrow anatomical niche within 25 μm of the epithelium, where the pH is near neutral. Here, *H*. *pylori* live either as a free-swimming population within the protective mucus layer, or they attach to the epithelial surface, where they persist as cell-associated microcolonies [4–8]. Attached bacteria extract nutrients from gastric cells to facilitate their growth on the epithelium [9,10].

Cell-associated *H*. *pylori* can also penetrate deeper into the mucosa and persist as microcolonies on the epithelial surface of the gastric glands [11,12]. The midzone and base of the gland is a specialized microenvironment, or “microniche,” within the stomach that contains epithelial precursor and stem cells. The gland-associated bacteria have profound effects on host biology, as they alter gastric stem cell proliferation and induce inflammation and hyperplasia [13]. However, the advantage of colonizing the gland microniche is unclear. Here, we present novel techniques to observe and quantify the dynamics of *H*. *pylori* gland colonization in vivo in a murine model of chronic infection, and how this process is controlled by host factors.

We generated isogenic wild-type (WT) and mutant *H*. *pylori* strains that are differentially tagged with fluorophores to study the dynamics of gland-associated bacteria in a murine model of chronic infection. Using quantitative three-dimensional (3D) confocal microscopy and passive CLARITY technique (PACT) to analyze intact stomachs infected with differentially tagged *H*. *pylori* strains, we found that the bacteria establish distinct populations within patches of gastric mucosa comprised of hundreds of glands. These clonal populations arise from a small number of founder bacteria, expand to adjacent glands over time, and persist throughout chronic infection. These data suggest that gland-associated *H*. *pylori* serve as stable bacterial reservoirs that may reseed the more transient populations in the surface mucosa. Furthermore, *H*. *pylori* gland populations exhibit intraspecies colonization resistance by excluding isogenic competitors from this microniche. Mutants unable to colonize the glands cannot exert colonization resistance, and knockdown of the gland population with antibiotics restores the availability of this niche for incoming bacteria. Finally, we characterized host factors that regulate *H*. *pylori* gland colonization, and show that both the age at infection and T-cell responses impact the density of gland-associated bacteria during chronic infection. Collectively, our work demonstrates that *H*. *pylori* infection of the gastric mucosa is not homogeneous. Rather, establishing stable populations within the gland microniche generates a long-term reservoir for persistence and colonization resistance.

## Results and discussion

### *H*. *pylori* establish stable population islands in the gastric glands

To understand how gland-associated *H*. *pylori* populations establish themselves and contribute to gastric colonization, we generated differentially labeled, isogenic WT bacterial strains in the mouse-adapted *H*. *pylori* PMSS1 background to visualize gland colonization in vivo. To do so, we expressed green fluorescent protein (GFP) [14] or tdTomato (tdT) [15] under control of the *ureA* promoter at the *rdxA* chromosomal locus, generating *Hp* GFP and *Hp* tdT, respectively (S1A and S1B Fig). Both strains are equally fit in terms of in vitro growth, attachment to gastric epithelial cells, and translocation of the CagA effector into host cells (S1C–S1E Fig). Because the *H*. *pylori* genome varies extensively, even between single colonies isolated from the same WT stock [16], we performed whole genome sequencing to assess differences between the *Hp* GFP and *Hp* tdT genomes. We found that these genomes were highly similar (99.98% nucleotide sequence identity). Single nucleotide polymorphisms (SNPs) were identified in seven loci, but only in two genes did they predict a nonconservative amino acid change (S1 Table). Comparison of the genomes of our PMSS1 WT stock with the published strain revealed 99.99% sequence identity, suggesting that differences between *Hp* GFP and *Hp* tdT fall within the range of variation seen for WT isolates.

**Fig 1:**
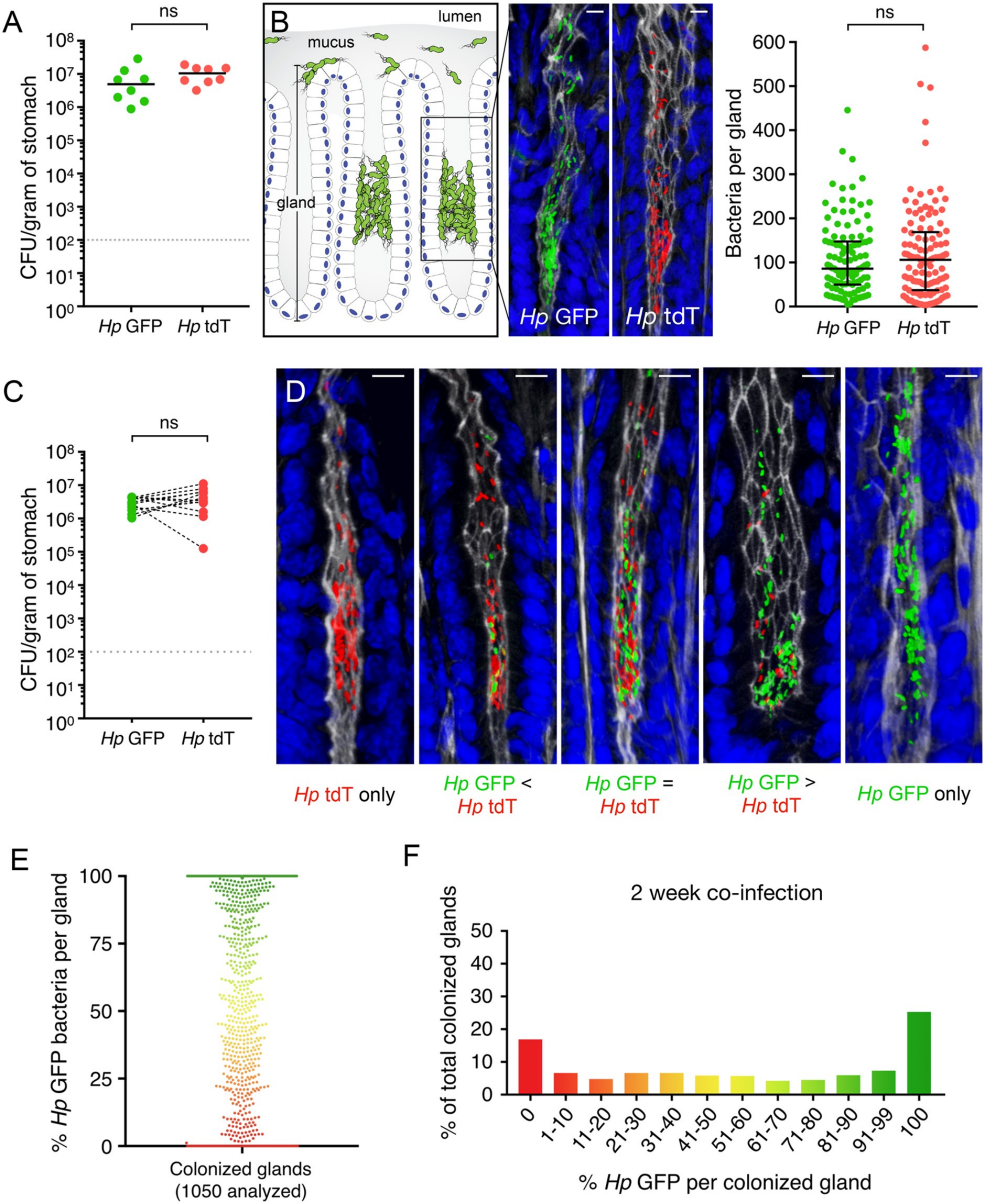
Bacterial populations within individual glands are heterogeneous. (A) Recovered CFU per gram of stomach at 2 weeks post-infection with *Hp* GFP or *Hp* tdT (eight mice per group). Data represent two independent experiments. Gray dotted line, limit of detection; black bars, median. Relevant data values are included in S1 Data. (B) Left, cartoon schematic of the gastric epithelium. The gastric epithelium consists of a single layer of columnar epithelial cells that is organized into repeated, invaginated units called glands. The epithelium is covered with a protective mucus layer (gray) that shields the underlying cells from the acidic lumen. *H*. *pylori* (green) reside within the surface mucus in a free-swimming state, or directly attached to the surface epithelium and the epithelium deep in the gastric glands. The gland-associated bacteria are concentrated in the mid-glandular region, which contains gastric epithelial precursor cells. The mid-glandular region is indicated by the inset, which zooms into a 3D confocal image of *H*. *pylori* within a gland. Middle, 3D confocal images of gland-associated bacteria in *Hp* GFP–or *Hp* tdT–infected mice from panel A. Nuclei (blue), F-actin (white), GFP (green), tdT (red); scale bar, 10 μm. Right, 113 total colonized glands from three mice per group analyzed to determine the number of bacteria per gland. Black bars, median; error bars, interquartile range. Relevant data values are included in S1 Data. (C) Total CFU/g recovered from mice co-infected with *Hp* GFP and *Hp* tdT at 2 weeks post-infection (12 mice). Black dashed lines connect *Hp* GFP and *Hp* tdT counts recovered from the same mouse. Data represent three independent experiments. Gray dotted line, limit of detection. Relevant data values are included in S1 Data. (D) Three-dimensional confocal images of individual gastric glands from co-infected mice from panel C. Nuclei (blue), F-actin (white), GFP (green), tdT (red); scale bar, 10 μm. (E) Percent *Hp* GFP bacteria per gland (1,050 colonized glands analyzed from seven co-infected mice). Each dot represents a colonized gland. (F) Data from panel E plotted as a histogram. The x-axis represents percentage of *Hp* GFP bacteria per colonized gland, separated into 12 bins; the y-axis represents percentage of analyzed glands that are present in each bin. Relevant data values are included in S1 Data. Statistics: *p*-values obtained using Mann–Whitney test (panels A,B) or Wilcoxon signed-rank test (panel C). CFU, colony-forming unit; GFP, green fluorescent protein; NS, no significance; tdT, tdTomato; 3D, three-dimensional.

After demonstrating equal fitness of *Hp* GFP and *Hp* tdT in vitro, we assessed the ability of these isogenic strains to colonize our mouse infection model. When inoculated into mice individually, both strains infected to similar levels and colonized the gastric glands (Fig 1A and 1B). To determine if the two strains are equally fit in competition, we orally inoculated mice with 10^8^ colony-forming units (CFU) of an equal mixture of *Hp* GFP and *Hp* tdT. Both *Hp* GFP and *Hp* tdT colonized mice to similar numbers at 2 weeks post-infection (Fig 1C). Because *H*. *pylori* are motile and intermix in the mucus (S1F Fig) [7,17,18], we hypothesized that mixed populations would continuously enter and exit gastric glands. We thus expected to find equal mixtures of green and red bacteria in each gland. Instead, 3D confocal reconstructions of co-infected stomachs revealed that individual glands contained unique ratios of *Hp* GFP to *Hp* tdT, ranging from a 50:50 mixture to pure populations of only one strain (Fig 1D). Quantitative analysis of *Hp* GFP to *Hp* tdT ratios per colonized gland showed that glands containing a single strain are more prevalent than those with equal mixtures (Fig 1E and 1F). This supports alternative models for the establishment and kinetics of gland-associated bacteria. One possibility is that each gastric gland is a discrete niche populated by a unique mix of founder bacteria. In this case, we would expect different *Hp* GFP:*Hp* tdT gland ratios to be randomly distributed throughout the tissue, depending on which founders happened to arrive first. Alternatively, if a higher level of organization of these distinct gland ratios exists, we would expect specific ratios of green and red bacteria to group together spatially within the tissue.

**Fig 2:**
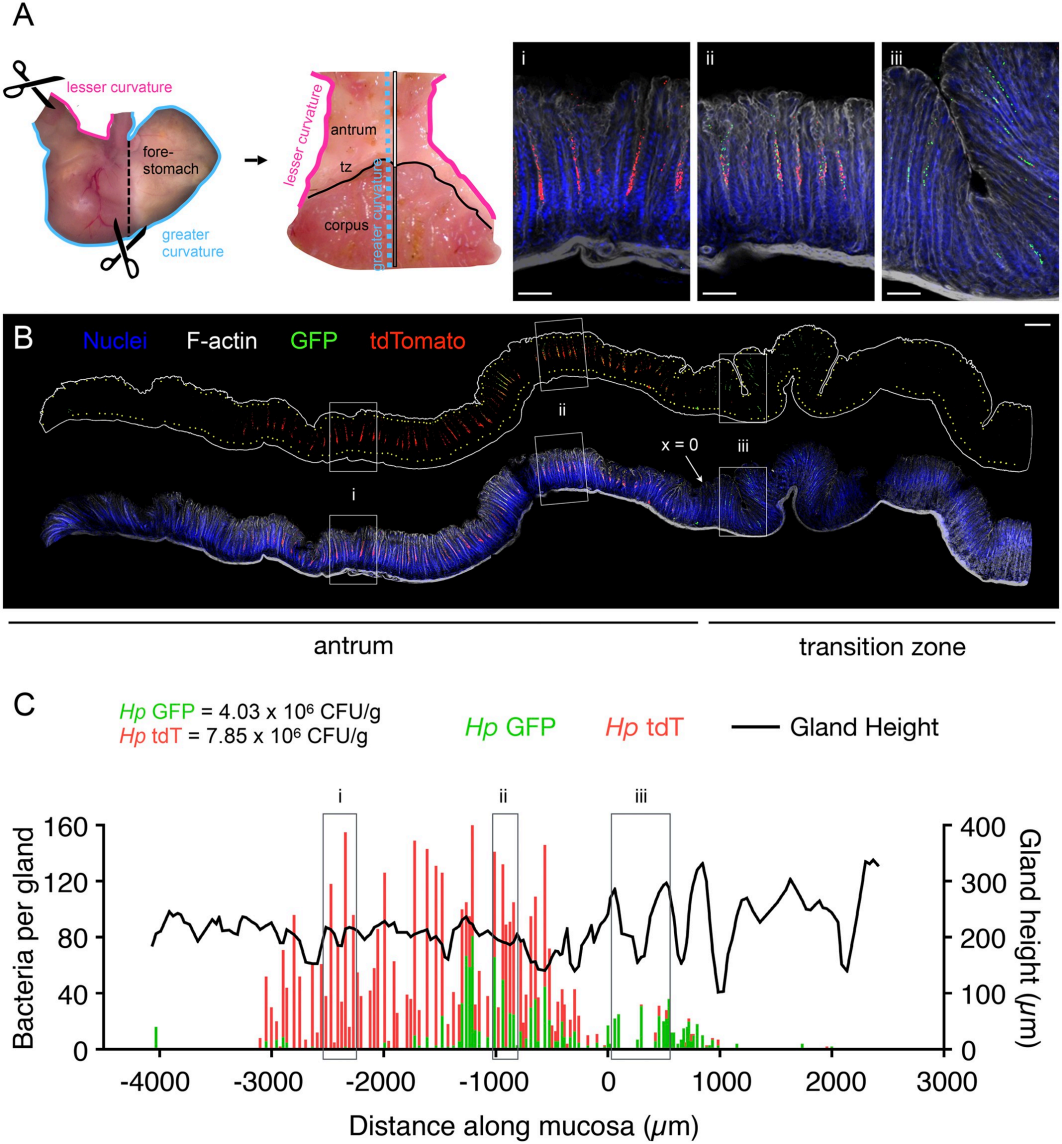
*H*. *pylori* gland populations are organized into patches within the mucosa. (A) Schematic of stomach sectioning. Mouse stomachs were opened along the lesser curvature and flattened. The corpus, transition zone (tz, solid black line), and antrum and the lesser (magenta line) and greater (blue line) curvatures are identified. Longitudinal sections were taken along the greater curvature and include all gastric regions. (B) Image of longitudinal section from 2-week co-infected mouse. x = 0 is the junction between the antrum and transition zone. For clarity, top image shows bacterial signal without nuclei and F-actin overlaid. Yellow dots mark the top and base of each gland (scale bar, 150 μm). Boxes i, ii, and iii are magnified in the upper right corner of the figure (scale bar, 50 μm). (C) Gland height (black line) and bacteria per gland (green and red bars) are mapped according to their location within the longitudinal section in panel B. Boxes i, ii, and iii correspond to boxed regions in panel B. Total CFU/g of each strain recovered from this mouse indicated. Relevant data values included in S1 Data. CFU, colony-forming unit; GFP, green fluorescent protein; tdT, tdTomato; tz, transition zone.

To determine how these heterogeneous gland populations are organized relative to each other, we took 150-μm-thick longitudinal sections (10–15 mm long) through the greater curvature of the co-infected stomachs, to include antrum, transition zone, and corpus regions, and processed these samples for quantitative confocal microscopy (Fig 2A). We obtained high-resolution images of the sections, tiled the overlapping scanned regions, and mapped the location and number of *Hp* GFP and *Hp* tdT within individual glands along longitudinal space (S1 Movie). Most bacteria were found in the antrum and transition zone, with very few to no bacteria within the corpus glands, as we previously showed for *H*. *pylori* PMSS1 [13,19]. Mapping analysis of the antrum and transition zone revealed that distinct ratios of *Hp* GFP:*Hp* tdT in individual glands are not randomly distributed but rather are organized into 1–2-mm regions, where one region may contain a unique strain next to areas with mixed ratios (Fig 2). This regional distribution holds true across multiple co-infected mice, although the regional patterns are unique when comparing different mice and even in different sections from the same mouse (S2 Fig).

**Fig 3:**
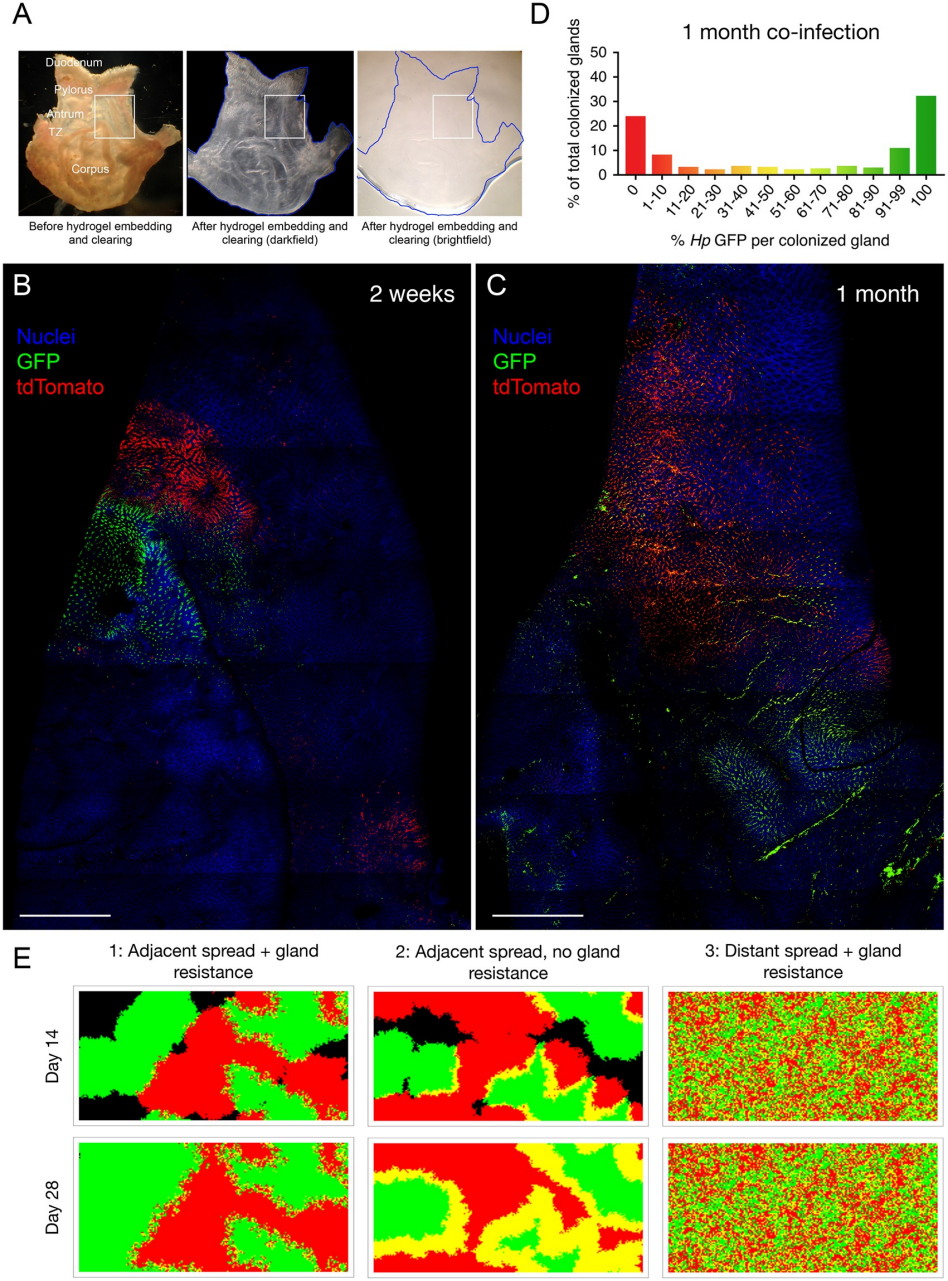
PACT reveals that gland-associated *H*. *pylori* form stable population islands. (A) Mouse stomach processed through PACT. Before hydrogel embedding and clearing via SDS, the stomach is opaque. After, it is optically transparent. Scale box, 4.9 × 5.3 mm. (B, C) Images of glands from mice co-infected with *Hp* GFP and *Hp* tdT, shown from a top-down view. Images from mouse infected as adult at 2 weeks post-infection (B) or infected as neonate at 1 month post-infection (C). Scale bar, 1 mm. (D) Percentage *Hp* GFP bacteria per gland in co-infected mice at 1 month post-infection (300 colonized glands analyzed from three mice, includes one infected as adult and two infected as neonates). Relevant data values are included in S1 Data. (E) Representative examples of computer simulations of gland colonization and spread. Ten randomly selected glands were colonized by *Hp* GFP and another set of ten by *Hp* tdT in a field of 20,000 available glands, and bacteria were allowed to spread over time. Green, *Hp* GFP–occupied glands; red, *Hp* tdT–occupied glands; yellow, 50:50 co-occupied glands; black, unoccupied. Three different colonization scenarios were simulated, including or excluding parameters of adjacent spread or gland resistance. GFP, green fluorescent protein; PACT, passive CLARITY technique; SDS, sodium dodecyl sulfate; tdT, tdTomato; TZ, transition zone.

The observed strain distributions along longitudinal space suggest that gland-associated *H*. *pylori* may be organized as clonal patches in 3D regions of the mucosa. To evaluate *H*. *pylori* distribution in 3D space, we utilized PACT, a method that renders tissues optically transparent and allows for imaging of intact organs [20,21] (Fig 3A). We imaged clarified stomach tissue from the mucosal surface and into the glands using a 10× objective to capture a global view of the distribution of colonized glands. By tiling 30–40 fields of view, we were able to map regions of the antrum and transition zone containing 10,000–20,000 glands (20–40 mm^2^) with resolution down to single gland units (S2 Movie). We observed that gland-associated *H*. *pylori* indeed form distinct population islands. By comparing regions of only one strain color to adjacent regions with mixed strains, we found that these islands consist of between 250 and 1,500 glands at 2 weeks post-infection (Fig 3B).

These findings suggest that *H*. *pylori* form clonal population islands in the gastric glands that do not rapidly mix. To understand the kinetics of mixing, we compared the distribution of *H*. *pylori* population islands at 2 weeks post-infection with animals co-infected with both strains for 1 month (Fig 3C and S5 Fig). We analyzed mice infected as adults or as neonates, because bacteria persist at approximately 10-fold higher densities during chronic stages of infection in animals colonized as neonates due to the development of immunological tolerance [22]. We expected that if gland populations gradually intermix, the *Hp* GFP:*Hp* tdT ratio per gland would become more homogenous over time (50:50 *Hp* GFP:*Hp* tdT). Instead, we found that the gland-associated population islands expanded in size but remained distinct at 1 month post-infection (in mice infected as adults or neonates). Furthermore, the *Hp* GFP:*Hp* tdT ratios per colonized gland did not become more homogeneous (Fig 3C and 3D). This suggests that gland-associated population islands do not intermix over the course of a chronic infection, implying their role as stable bacterial reservoirs. Such a reservoir may reseed the more transient populations in the superficial mucosa, which are likely lost daily due to gastric mucus turnover, epithelial cell shedding, and peristalsis.

The island-like distribution of *H*. *pylori* populations suggests a model in which founder bacteria seed regions of susceptible mucosa early in the colonization period, and then spread to nearby glands to form patches where they persist during chronic infection. Indeed, despite an inoculum of 10^8^ CFU, only approximately 100 bacteria were recovered from a whole stomach at 6 hours post-infection (S3A Fig). Thus, only a minute fraction of the infectious dose establishes colonization. A previous study analyzing colonized glands by dissociating the tissue into single gland units similarly reported that few glands are initially occupied by *H*. *pylori*, but an increased number of glands become colonized over time [12]. However, our systematic mapping of intact stomachs via PACT enabled us to uncover a unique pattern of mucosal colonization and spread not observable otherwise. As early as 3 days post-infection, we could detect distinct patches composed of between 1 and 15 glands in longitudinal gastric sections, which increased in size over 2 weeks of infection (S3B Fig). Using PACT to analyze 5 day co-infected stomachs, we observed that these bacterial population islands start as small, distinct patches consisting of a single color (250–500 glands) (S3C and S3D Fig). Together, these results indicate that an inoculated population experiences a dramatic bottleneck during colonization, and that very few bacterial founders establish infection within glands. We propose that these gland-associated bacteria expand locally as patches by infecting adjacent glands until a majority of permissive gland niches are occupied. Islands consisting of a mixture of both strains may result either from the overlap of single-colored patches as they spread to unoccupied glands, or from two opposite-colored bacteria initially seeding the same gland.

To test these interpretations of our data, we conducted computer simulations to visualize how different modes of colonization and spread would shape the distribution of gland-associated *H*. *pylori* islands in co-infected stomachs. We began all simulation runs by allowing 10 individual bacteria per strain to establish randomly in a region containing 20,000 glands. Next, we allowed the bacteria to spread throughout the given region for a total of 28 days, with the following parameters: (a) the bacteria can either only spread from occupied glands to unoccupied glands that are directly adjacent (adjacent spread) or can spread to any unoccupied glands regardless of proximity (distant spread), and (b) once a gland is colonized to saturation, that population remains in that gland and excludes incoming bacteria (gland resistance), or glands remain receptive for new bacteria to enter and intermix. We used a simplified model in which only three gland types could arise: *Hp* GFP occupied, *Hp* tdT occupied, or a 50:50 mixture of both. Our murine co-infection results show that the majority of glands are composed of a single strain and form large population islands that are stable over time. The simulation scenario that best reproduced our experimental data was the one with both adjacent spread and gland resistance (Fig 3E and S4 Fig, Scenario 1). If we eliminated gland resistance and assumed that bacteria can constantly enter and colonize glands, single-colored patches did not persist and instead became more homogeneously mixed over time (Fig 3E and S4 Fig, Scenario 2). If we allowed *H*. *pylori* to spread to any vacant glands in the stomach (not just adjacent ones), this resulted in an increased number of co-occupied glands and a loss of large, distinct single-colored patches (Fig 3E and S4 Fig, Scenario 3).

### *H*. *pylori* establishes colonization resistance against incoming isogenic strains early in infection

**Fig 4:**
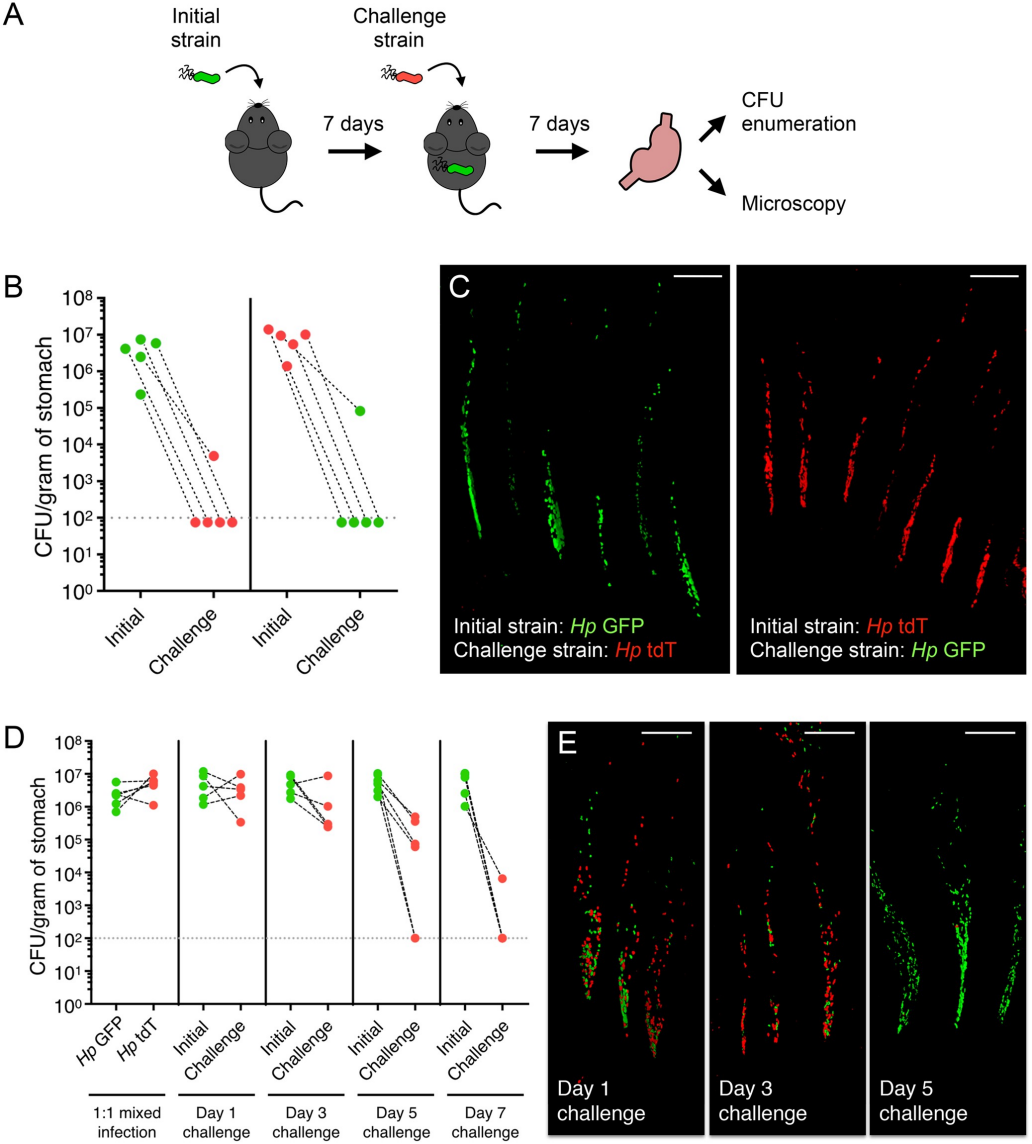
*H*. *pylori* exerts intraspecies colonization resistance early in infection. (A) Sequential infection schematic. Mice were infected with the initial strain for 1 week, challenged with the opposite-colored isogenic strain, and stomachs were harvested after 1 additional week for CFU enumeration of both initial and challenge strains and visualization of gland colonization by microscopy. As an example, *Hp* GFP was depicted as the initial strain and *Hp* tdT as the challenge strain in this diagram. However, the reverse experiment, in which *Hp* tdT was the initial strain and *Hp* GFP was the challenge strain, was also conducted. (B) Total CFU/g recovered from sequentially infected mice as depicted in panel A. Both iterations in which *Hp* GFP was either the initial strain (left) or challenge strain (right) are shown (five mice per group). Black dashed lines connect *Hp* GFP and *Hp* tdT counts recovered from the same mouse at 2 weeks post-inoculation of the initial strain. Gray dotted line, limit of detection. Relevant data values are included in S1 Data. (C) Three-dimensional confocal images of bacteria in glands from sequentially infected mice in panel B. GFP (green), tdT (red); scale bar, 30 μm. (D) Total CFU/g recovered from mice pre-colonized with *Hp* GFP for different time points (1, 3, 5, or 7 days) prior to challenge with *Hp* tdT (5–6 mice per group). Stomachs were harvested at 2 weeks post-inoculation of the initial strain for CFU enumeration of both initial and challenge strains. Data represent two independent experiments. Black dashed lines connect *Hp* GFP and *Hp* tdT counts recovered from the same mouse. Gray dotted line, limit of detection. Relevant data values are included in S1 Data. (E) Three-dimensional confocal images of bacteria in glands from sequentially infected mice in panel D, 2 weeks post-inoculation of the initial strain. GFP (green), tdT (red); scale bar, 30 μm. CFU, colony-forming unit; GFP, green fluorescent protein; tdT, tdTomato.

Our data so far suggest that once *H*. *pylori* establish population islands in the glands, they remain stable throughout chronic infection and do not mix with new incoming bacteria. In the ecology literature, community assembly is known to be influenced by priority effects, a phenomenon in which early colonization of a niche confers an advantage for one member against future colonizers [23]. Several studies have shown that priority effects govern bacterial colonization in the gastrointestinal tract, such as the pathogen *Salmonella* [24] or the gut commensal *Bacteroides* [25,26]. To determine if the order of arrival dictates who populates the gastric glands, we used a sequential infection model in which one isogenic strain was allowed to colonize and establish in the glands for a week before introducing the alternate-colored strain. After another week, the stomachs were harvested for CFU enumeration (Fig 4A). We found that if isogenic, fluorescent *H*. *pylori* strains were sequentially introduced, the challenge strain did not establish in the stomach (Fig 4B). Furthermore, only the initial strain was found in the glands, with none of the challenge strain present (Fig 4C). This indicates that an initial infection by *H*. *pylori* prevents subsequent super-colonization by an isogenic strain, consistent with previous studies demonstrating self-colonization resistance in other *H*. *pylori* strains [12,27].

To determine when colonization resistance is established and if it correlates with gland occupation, we introduced the challenge strain (*Hp* tdT) at various time points before the initial strain (*Hp* GFP) could overtake the majority of gland niches. We found that the challenge strain could colonize the stomach and establish in the glands when introduced 1–3 days after the initial strain. However, if the pre-colonized mice were infected with the challenge strain 5–7 days after the initial strain, the challengers were excluded from the glands (Fig 4D and 4E). These results indicate that colonization resistance correlates with increased occupation of the gastric glands.

### Gland occupation is important for *H*. *pylori* intraspecies colonization resistance

**Fig 5:**
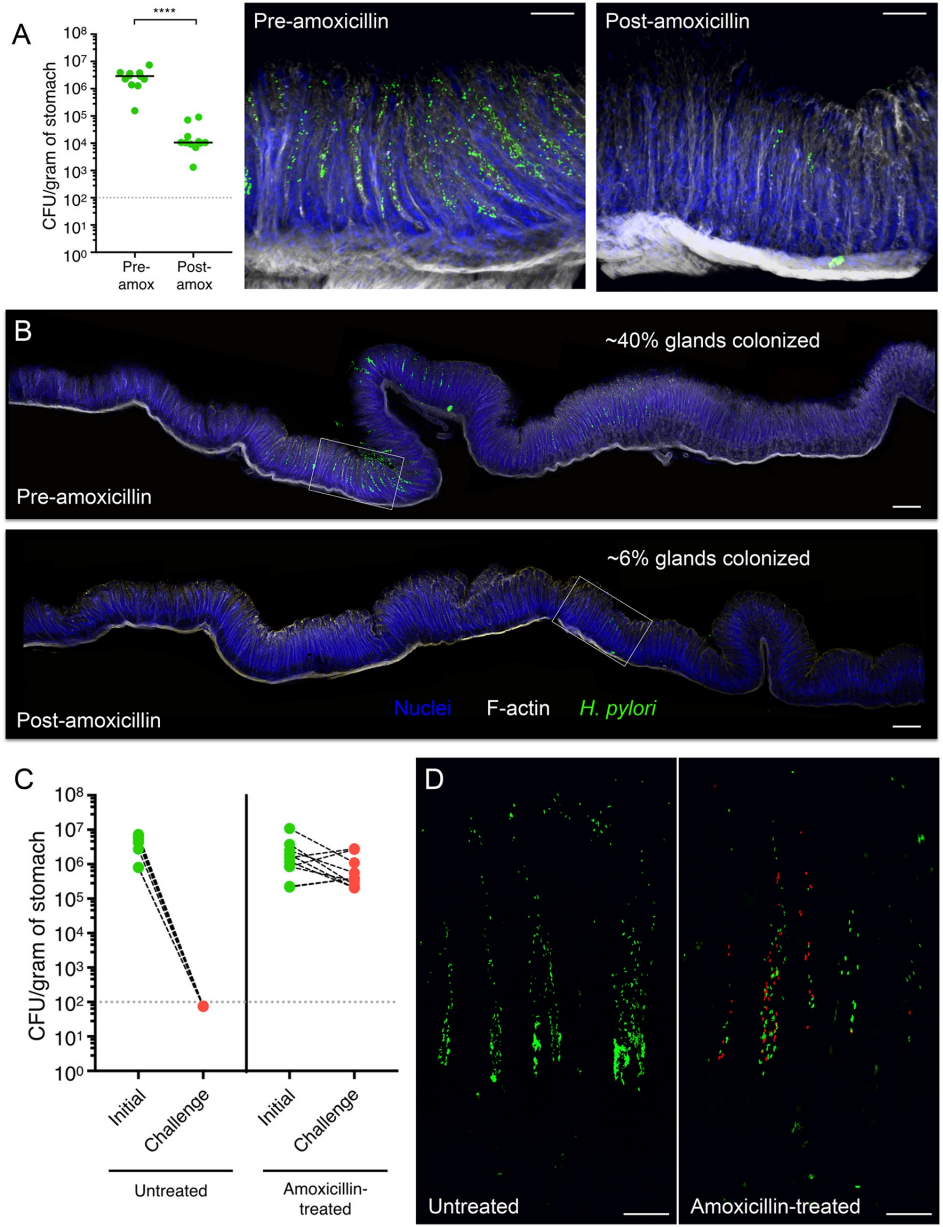
Knockdown of pre-established gland population with antibiotics abolishes colonization resistance. (A) Left, total CFU/g recovered from mice colonized with *Hp* GFP for 1 week (pre-amoxicillin), and then treated with amoxicillin for 3 days (post-amoxicillin) (11 mice per group). Gray dotted line, limit of detection; black bars, median. Data represent three independent experiments. Statistics: *p*-value obtained using Mann–Whitney test. ****p < 0.0001. Right, magnified images of regions indicated by insets from panel B. Scale bar, 50 μm. Relevant data values are included in S1 Data. (B) Images of longitudinal stomach sections from mice in panel A. Regions indicated by insets are magnified above. Percentage of antral and transition zone glands colonized indicated for each individual mouse. Scale bar, 75 μm. (C) Total CFU/g recovered from mice pre-colonized with *Hp* GFP for 1 week, given water or water infused with amoxicillin for 3 days, and challenged with *Hp* tdT. After 1 additional week, stomachs were harvested for CFU enumeration of the initial and challenge strains (10 mice per group). Black dashed lines connect *Hp* GFP and *Hp* tdT counts recovered from the same mouse. Gray dotted line, limit of detection. Data represent two independent experiments. Relevant data values are included in S1 Data. (D) Three-dimensional confocal images of bacteria in glands from sequentially infected mice in panel C. GFP (green), tdT (red). Scale bar, 30 μm. CFU, colony-forming unit; GFP, green fluorescent protein; tdT, tdTomato.

Colonization resistance between strains in other microbes is caused by occupation of a specific spatial niche [24,25], nutritional restriction, production of antimicrobials, and/or adaptation to immunological challenges [28,29]. In our model, colonization resistance is established before there is histological evidence of inflammation and correlates with increased occupation of the glands. Given this association, we used two methods to perturb the established gland population to see if priority effects are abolished. First, we treated pre-colonized mice with a subtherapeutic dose of amoxicillin for 3 days, which reduced bacterial numbers approximately 100-fold without clearing infection, but it was sufficient to eliminate most of the gland-associated bacteria (Fig 5A and 5B). When mice pre-colonized with *Hp* GFP and antibiotic treated were challenged with *Hp* tdT, colonization resistance was abolished. Furthermore, the challenge strain established in the stomach and colonized the glands in antibiotic-treated mice, but not in untreated controls (Fig 5C and 5D). This suggests that colonization resistance is reversible. However, antibiotic treatment not only reduces the gland-associated bacteria but also affects the mucus population. Hence, we cannot distinguish if the loss of priority effects after antibiotic treatment is due to increased availability of gland niches or to reduction of total bacterial burden.

**Fig 6:**
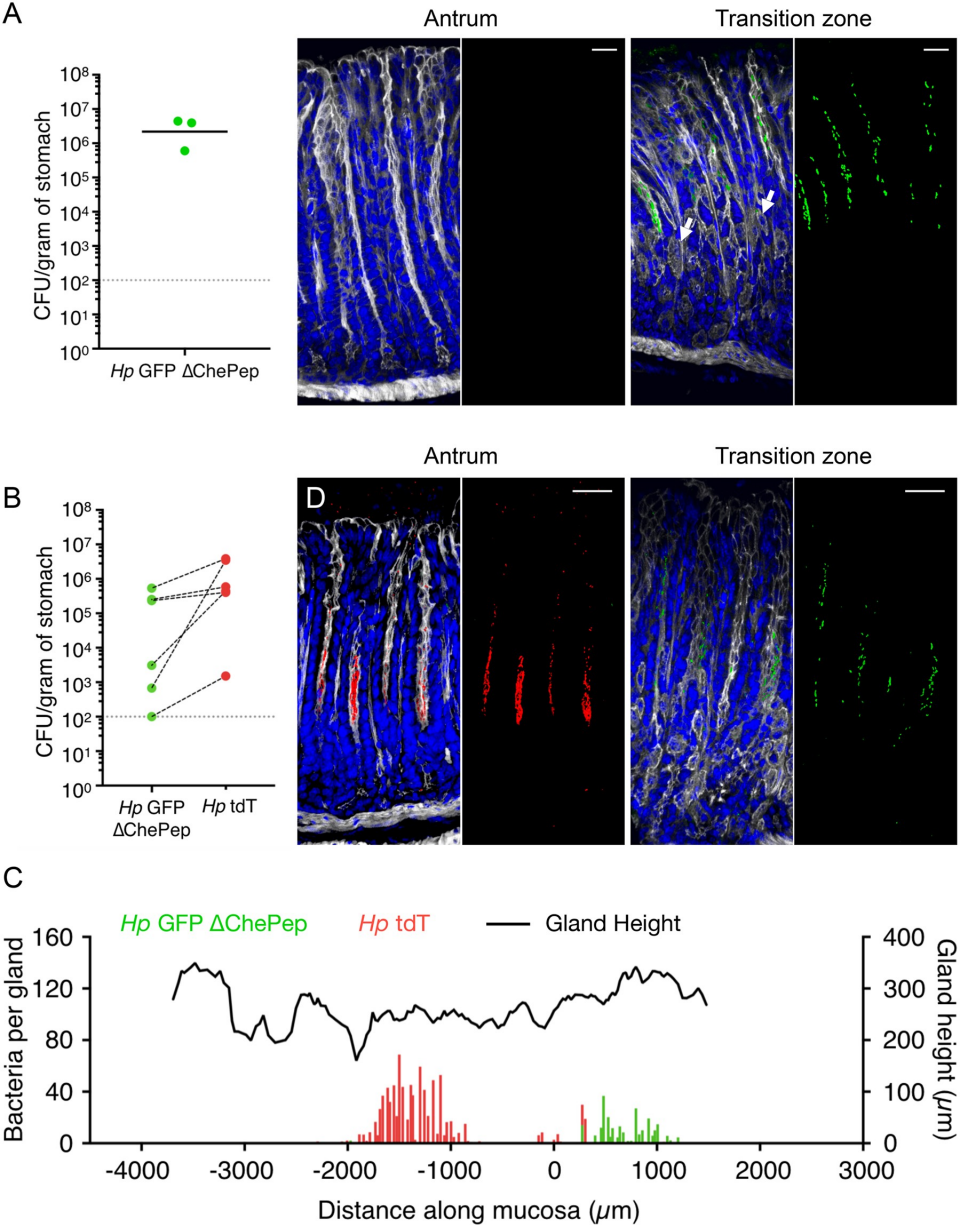
A bacterial mutant that does not colonize the antral glands cannot exert colonization resistance. (A) Left, total CFU/g recovered from mice infected with *Hp* GFP Δ*chePep* for 1 week (three mice). Gray dotted line, limit of detection; black bars, geometric mean. Right, Three-dimensional confocal images of antral or transition zone glands from infected mice. The transition zone is characterized by the presence of parietal cells in the bottom third of gastric glands (arrows). Nuclei (blue), F-actin (white), *H*. *pylori* (green); scale bar, 30 μm. Relevant data values are included in S1 Data. (B) Total CFU/g recovered from mice pre-colonized with *Hp* GFP Δ*chePep* for 1 week and then challenged with *Hp* tdT. Stomachs were harvested at 2 weeks post-inoculation of the initial strain for CFU enumeration of initial and challenge strains (six mice). Black dashed lines connect *Hp* GFP Δ*chePep* and *Hp* tdT counts recovered from the same mouse. Gray dotted line, limit of detection. Data represent two independent experiments. Relevant data values are included in S1 Data. (C) Mapping of location and number of gland-associated bacteria across a longitudinal stomach section of a mouse from panel B. Gland height (black line) and bacteria per gland (green or red bars) are plotted. x = 0 is the junction between the antrum and transition zone. Total CFU/g for each strain recovered from this mouse: *Hp* GFP Δ*chePep* = 5.36 × 10^5^ CFU/g, *Hp* tdT = 3.87 × 10^6^ CFU/g. Relevant data values are included in S1 Data. (D) Three-dimensional confocal images of antral or transition zone glands from the longitudinal section analyzed in panel C. GFP (green), tdT (red); scale bar, 30 μm. CFU, colony-forming unit; GFP, green fluorescent protein; tdT, tdTomato.

To selectively target the gland population and determine its role in priority effects, we used an *H*. *pylori* mutant in the chemotaxis regulator protein ChePep, which we previously described to be deficient in gland colonization. Although Δ*chePep* infected mice to similar levels as WT bacteria, the mutant could not colonize glands in the gastric antrum [11]. We verified these findings using our fluorescent strains. However, a more systematic mapping of bacterial distribution revealed that Δ*chePep* colonized a few glands in the transition zone but was absent from antral and corpus glands (Fig 6A). To test if Δ*chePep* exerts colonization resistance, we pre-colonized mice with a GFP-labeled Δ*chePep* mutant (*Hp* GFP Δ*chePep*) and allowed it to establish for 1 week before introducing *Hp* tdT. We found that mice pre-colonized with *Hp* GFP Δ*chePep* lacked colonization resistance and that the challenging *Hp* tdT strain was able to establish in the stomach (Fig 6B). Furthermore, mapping bacterial location within the glands of these samples revealed that *Hp* GFP Δ*chePep* colonized the transition zone glands, while *Hp* tdT colonized the antral glands that *Hp* GFP Δ*chePep* were unable to occupy (Fig 6C and 6D).

Together, these sequential infection experiments provide evidence for strong priority effects on *H*. *pylori* populations in the stomach. The ability of initial colonizers to exert colonization resistance against incoming challengers is associated with gland occupation, suggesting that the order in which *H*. *pylori* strains arrive and inhabit the glands determines the outcome of competitive interactions. Indeed, longitudinal clinical studies monitoring *H*. *pylori*–infected patients showed that individuals were colonized by the same strain(s) for years [30–33], suggesting that once an *H*. *pylori* strain is established in a stomach, it persists in that host for life and resists colonization by other strains.

### Host T-cell responses control *H*. *pylori* density in the gastric glands

Previously, we documented that sites of gland colonization correlate with regions of mucosal inflammation, suggesting that gland-associated bacteria induce host inflammatory responses [13]. Moreover, studies in both humans and mice have shown that the type of T-cell response induced by *H*. *pylori* infection determines the degree of gastritis and overall bacterial burden in the stomach. For instance, mice colonized as young adults (5–8 weeks old) mount a T-helper 1 (Th1)-biased CD4+ T-cell response, in which the production of interferon γ (IFNγ) and other pro-inflammatory cytokines leads to the development of gastritis and a decrease in bacterial burden starting at 1 month post-infection [22,34,35]. Similar observations were made in infected adult patients [36,37]. In contrast, children or mice colonized as neonates (1 week old) retain higher bacterial levels even at later time points due to the development of immunological tolerance, in which the induction of regulatory T cells suppressed CD4+ effector T-cell responses and led to less bacterial clearance and immunopathology [22,38–40]. Given these findings, we hypothesized that tolerogenic versus pro-inflammatory T-cell responses may control bacterial density within the glands during chronic infection.

**Fig 7:**
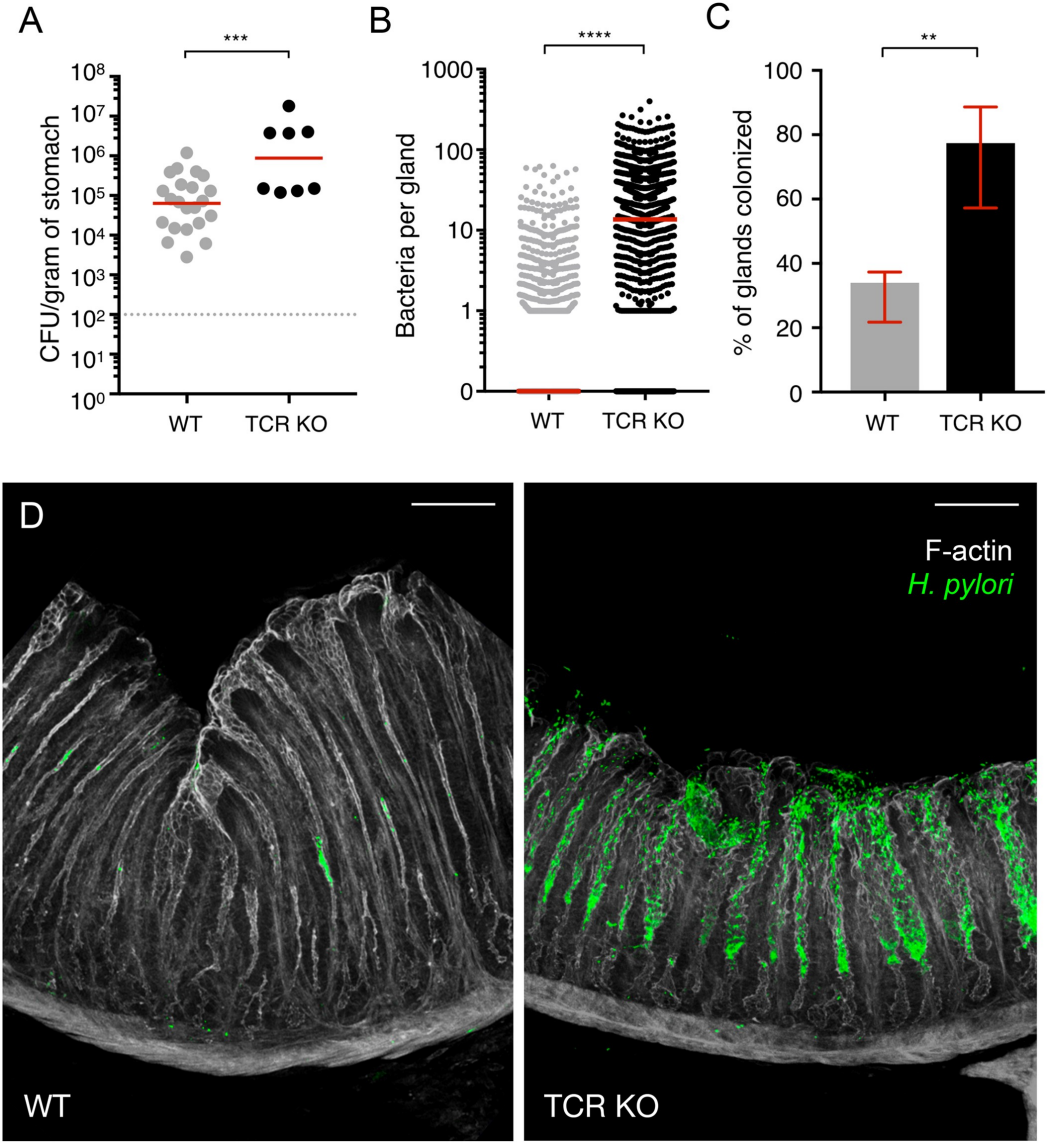
Host T cells impact the density of gland-associated bacteria. (A) Total CFU/g recovered from WT or TCRβ/δ^−/−^ (TCR KO) mice infected with *H*. *pylori* PMSS1 WT for 8 weeks (8–22 mice per group). Gray dotted line, limit of detection; red bars, median. Data represent two independent experiments. Relevant data values are included in S1 Data. (B) Bacteria per gland in antrum and transition zone of WT or TCR KO mice at 8 weeks post-infection. A total of 200–300 glands were analyzed per mouse for both WT and TCR KO groups (five mice per group). Red bars, median. Relevant data values are included in S1 Data. (C) Percentage of colonized glands in the antrum and transition zone of WT or TCR KO mice at 8 weeks post-infection. Bars, median; error bars, interquartile range. Relevant data values are included in S1 Data. (D) Images of antrum of WT or TCR KO mice at 8 weeks post-infection. Scale bar, 80 μm. Statistics: *p*-values obtained using Mann–Whitney test (panels A–C). **p < 0.01, ***p < 0.001, ****p < 0.0001. CFU, colony-forming unit; KO, knockout; TCR, T-cell receptor; WT, wild-type.

To determine how the host’s age at infection and the corresponding T-cell responses affect the gland-associated bacteria, we infected mice as adults or neonates with *H*. *pylori*. At 1 month post-infection, mice infected as neonates contain approximately 10 times higher overall bacterial levels, as previously observed, and also higher numbers of bacteria per gland compared with mice infected as adults (S5 Fig). This suggests that host T-cell responses not only impact total CFU burden but also bacterial density within the gastric glands. Given these data, we predicted that adult mice with impaired effector T-cell function would maintain a high density of gland-associated bacteria. To test this, we infected adult WT mice or TCRβ/δ^−/−^ mice (which lack functional T cells) [41] with *H*. *pylori* and harvested stomachs at 8 weeks post-infection. At this time point, the overall bacterial burden was about 10 times higher in TCRβ/δ^−/−^ mice compared with WT mice (Fig 7A). Examination of the gastric glands revealed that the number of colonized glands and bacteria number per gland are much higher in infected TCRβ/δ^−/−^ mice than WT mice (Fig 7B–7D).

Collectively, these results show that the host’s age at infection and the corresponding T-cell responses impact bacterial density within the gastric glands during chronic infection. Because *H*. *pylori* is usually acquired during childhood [2], it is plausible that induction of immunological tolerance and suppression of pro-inflammatory T-cell responses enable bacterial persistence in the glands and lifelong colonization.

### Conclusion

Since its discovery, *H*. *pylori* has been primarily thought to reside as a motile form in the mucus or attached to the epithelium in the superficial gastric pits [4,7]. However, the stomach mucosa is extremely dynamic. Gastric mucus is constantly secreted and renewed daily [42,43], and gastric contents are emptied every 4–5 hours [44]. Moreover, the gastric glands function like a conveyor belt. Precursor and stem cells constantly divide and differentiate, and terminally differentiated cells ascend the gland to reach the surface epithelium, where they senesce and are shed into the lumen every 2–3 days [45]. If its niche in the superficial mucosa is turned over daily, how does *H*. *pylori* survive for the life of its host? Previous studies proposed that *H*. *pylori* must constantly swim, attach to, and detach from the surface epithelium to avoid clearance by peristalsis and maintain a steady-state population [46,47]. Here, we propose that the gastric glands provide an additional protected microniche for *H*. *pylori* to maintain a chronic reservoir that can replenish the transient populations in the superficial mucosa—analogous to a “bacterial stem cell” population. It is likely that as gastric epithelial cells ascend the gland, a proportion of gland-associated bacteria are carried to the surface mucus and reseed the free-swimming population. Meanwhile, bacteria still adhered to the gland epithelium must continuously replicate to replenish the bacterial load lost through epithelial cell migration.

Similar to the gastric glands, the colonic crypts also serve as a critical niche for commensal microbes to maintain stable colonization in the murine gut [25,26,48]. When the colon was subjected to environmental disturbances (i.e., gut pathogens or antibiotic treatment), the bacterial commensal population in the lumen was decimated, whereas the crypt population survived [25]. This is intriguing given that intestinal commensal bacteria were previously thought to reside only within the lumen, separated from the colonic epithelium by a thick mucus layer [49,50]. Like our findings in the stomach, these studies highlight the significance of the crypt microniche in microbial persistence.

Quantitative analysis of longitudinal sections and of whole stomachs processed via PACT allowed us to map the location of *H*. *pylori* through an entire organ with unprecedented spatial resolution. This ability to analyze infection in situ, in the context of intact tissue architecture, provides critical information that is otherwise lost in population-level assays or when the tissue is homogenized prior to analysis. To date, only a few studies have used optical clearing methods in infectious disease research [51–53]. We believe that applying such tools holds great potential for uncovering novel insights into host–microbe interactions, in light of an increasing appreciation for the heterogeneity observed in infections across multiple pathogens [54–57]. Our findings via PACT suggest a new model of *H*. *pylori* mucosal colonization, in which a few founder bacteria initially seed regions of susceptible mucosa, replicate, and spread to neighboring glands. This raises questions about what signals *H*. *pylori* to spread to adjacent glands and how this is achieved. Because the gastric glands are a closed unit with a single opening at the gastric pits, bacteria reaching the surface mucus from a colonized gland need to swim back down and attach to cells in a nearby gland. The sensing of environmental signals may be involved in this process because *H*. *pylori* uses chemotaxis to navigate to favorable environments and avoid the acidic lumen [58]. Indeed, recent studies showed that deletion of the gene encoding the chemoreceptor TlpD, which is known to sense pH [19], reactive oxygen species (ROS) [59,60], and low energy–inducing environments [61,62], renders *H*. *pylori* unable to colonize as many glands as WT bacteria. However, this defect is partially rescued in mice treated with a proton pump inhibitor to lower acid production [19], or in mice that cannot generate ROS in the epithelium or in immune cells [59]. This suggests that sensing pH or ROS in the stomach may help *H*. *pylori* avoid unfavorable niches and efficiently spread to more glands. However, pH and ROS likely do not encompass all of the signals important for directing *H*. *pylori* to colonize adjacent glands and spread across the mucosa. For example, it is possible that energy taxis by TlpD could also play a role in *H*. *pylori* gland-to-gland spread and thus would be an interesting question to evaluate in the future. In addition, *H*. *pylori* detects the quorum sensing molecule autoinducer-2 (AI-2) as a chemorepellent, leading to bacterial detachment from surface-associated microcolonies [63,64]. Hence, it is possible that AI-2 may promote *H*. *pylori* detachment and spread to unoccupied glands.

Intriguingly, in our mouse model of infection, we never see complete occupation of 100% of the glands in the antrum and transition zone of the stomach, even when the bacteria reach maximum, steady-state levels. Our data from longitudinal sections (S2 Fig) reveal that at 2 weeks post-infection, the percentage of occupied glands in animals infected as adults varied from about 40% to 80% across different mice. Yet, all animals exhibited colonization resistance after 1 week of *H*. *pylori* infection. Similar observations have been documented in single glands isolated from *H*. *pylori*–infected stomachs at time points when bacterial numbers reach maximum levels [12]. Thus, we speculate that there is a certain percentage of glands that are not permissive for colonization, even though the physical space is technically “empty.” It is possible that these glands are intrinsically nonpermissive or that innate immune responses induced after *H*. *pylori* establishes in a set of glands restrict colonization of other parts of the tissue. Future studies to define the gland niche biochemically and structurally will uncover the differences between permissive and nonpermissive glands.

Localization to the gastric glands not only affects *H*. *pylori* persistence but also alters host biology. *H*. *pylori* colonizes the midzone and base of the glands, regions containing precursor and stem cells responsible for constant renewal of the gastric glands. We previously found that direct colonization of the glands by *H*. *pylori* induced local inflammation, glandular hyperplasia, and stem cell hyperproliferation, implying that microbial localization within tissues impacts host physiology [13]. Thus, understanding factors that control gland colonization will improve *H*. *pylori* clearance strategies to prevent pathology and disease. Here, we found that host factors such as the age at infection and T-cell responses control bacterial density within the glands during chronic infection. Future studies should explore additional bacterial, host, and environmental factors that impact colonization of this critical niche.

In summary, our work uncovered a novel mechanism of mucosal colonization, in which occupation of a specialized microniche establishes a stable bacterial reservoir, determines the pattern of colonization spread in the mucosa, and enhances bacterial persistence and colonization resistance. Understanding and targeting the microniches that house stable microbial reservoirs may allow us to eradicate mucosal colonizers associated with chronic disease or enhance long-term colonization of beneficial microbes.

## Materials and methods

### *H*. *pylori* strains and culture

*H*. *pylori* strain PMSS1 has been previously described [22]. All *H*. *pylori* strains used in this study were grown either on Columbia blood agar plates or in Brucella broth supplemented with 10% fetal bovine serum (FBS) (BB10) at 37 °C and 10% CO_2_, as previously described [65].

To generate GFP-expressing (*Hp* GFP) or tdTomato-expressing (*Hp* tdT) PMSS1, the entire open reading frame of *rdxA* was replaced by the respective fluorophore genes via natural transformation with linear constructs containing the *rdxA* flanking regions, the *aphA* gene (conferring kanamycin resistance), the *ureA* promoter, and the *egfp* or *tdTomato* gene. The *aphA* gene, *ureA* promoter, and *egfp* gene were amplified from the plasmid pTM115 that contains these components [66]. The *tdTomato* gene was amplified from the plasmid pYM50 [15]. These linear constructs were generated using a previously described PCR-based method [67]. Prior to natural transformation, the linear constructs were treated with PMSS1 cell-free extracts for in vitro methylation to increase transformation efficiency, as previously described [68], with a few modifications (see “In vitro methylation of linear DNA constructs” for more details). Primers used to generate fluorophore expression constructs are shown in S2 Table.

Fluorophore-expressing Δ*chePep* and Δ*cagE* mutants were generated by transforming previously published, nonfluorescent Δ*chePep* [11] and Δ*cagE* mutants [22] with genomic DNA from *Hp* GFP or *Hp* tdT.

### In vitro methylation of linear DNA constructs

Overnight broth cultures of *H*. *pylori* PMSS1 were subcultured and grown until mid-log phase. The bacterial cell pellet was resuspended in 5× volume of extraction buffer containing 20 mM Tris-acetate (pH 7.9), 50 mM potassium acetate, 5 mM Na_2_EDTA, 1 mM dithiothreitol (DTT), and a protease inhibitor cocktail (cOmplete, Mini, EDTA-free from Sigma-Aldrich, St. Louis, MO). The bacterial cell suspension was placed in a screw cap microcentrifuge tube containing 0.1-mm glass beads (Biospec Products, Bartlesville, OK). The tube was oscillated on a bead beater (Mini-Beadbeater-24 from Biospec Products) and then centrifuged at 15,000*g* for 5 minutes at 4 °C. The supernatant was removed, and protein concentration of the supernatant was determined using the Bio-Rad Protein Assay Kit II (Bio-Rad Laboratories, Hercules, CA). Linear DNA construct was combined with the supernatant (300–400 μg of protein) in a reaction containing extraction buffer (without protease inhibitor) and 200 μM S-adenosylmethionine (SAM) (New England BioLabs, Ipswich, MA), and incubated for 1 hour at 37 °C. Linear DNA construct was purified using the Zymo DNA Clean and Concentrator Kit (Zymo Research, Irvine, CA), and transformed into PMSS1.

### In vitro growth curves

Bacteria were grown for 24 hours in BB10 under microaerophilic conditions at 37 °C and then diluted to an OD_600_ of 0.05 to begin growth curve experiments. Samples were taken at 0, 4, 8, 12, 24, and 28 hours of growth, and the OD_600_ was measured to determine bacterial density. Three biological replicates were conducted per strain.

### Bacterial attachment assay

AGS cells (ATCC CRL 1739; human gastric adenocarcinoma epithelial cell line; ATCC, Manassas, VA) were cultured in Dulbecco’s Modified Eagle Medium (DMEM) (Gibco, Thermo Fisher Scientific, Waltham, MA) supplemented with 10% FBS (Gibco) and maintained at 37 °C in a 5% CO_2_ atmosphere. To assess whether *Hp* GFP and *Hp* tdT attach to gastric epithelial cells at the same levels, AGS cells were seeded to confluency in individual wells of a 24-well cell culture plate. *Hp* GFP or *Hp* tdT (approximately 10^8^ bacteria/mL) was added to individual wells containing confluent AGS cell monolayers, allowed to attach for 1 minute, and cell monolayers were washed with fresh DMEM to remove unattached bacteria. Subsequently, samples were harvested to determine the number of attached *H*. *pylori*. The monolayers were incubated with DMEM containing 1% saponin (which lyses host cells but not the bacteria) before scraping the monolayers off of the wells. The samples were then serially diluted and plated for CFU counts.

### Assessment of CagA delivery

To assess whether *Hp* GFP and *Hp* tdT deliver the effector protein CagA into host cells, AGS cells were seeded into six-well plates one day prior to use for infections. *Hp* GFP or *Hp* tdT (approximately 10^8^ bacteria/mL) was added to individual wells containing confluent AGS cell monolayers, allowed to attach for 5 minutes, and cell monolayers were washed two times with fresh DMEM to remove unattached bacteria. AGS cells were also infected with *Hp* GFP Δ*cagE* or *Hp* tdT Δ*cagE* as controls for no CagA delivery. Twenty-four hours after infection, cells were washed twice with fresh DMEM and fixed in 2% paraformaldehyde in 100 mM phosphate buffer (pH 7.4) at room temperature for 10 minutes. Cells were stained with appropriate antibodies and visualized by confocal microscopy to confirm cellular elongation in AGS cells infected with *Hp* GFP and *Hp* tdT, but not in AGS cells infected with *Hp* GFP Δ*cagE* or *Hp* tdT Δ*cagE*.

### Whole genome sequencing of *Hp* GFP and *Hp* tdT

DNA was sequenced on the Illumina HiSeq X platform using paired-end fragments of 450 bp, generating reads 150-bp long. Each genome was sequenced at approximately 300×. A Wellcome Sanger Institute in-house pipeline (https://github.com/sanger-pathogens/Bio-AutomatedAnnotation) was used for bacterial assembly and annotation. It carries out de novo assembly for each sequenced genome using Velvet v. 1.2.1032, SSPACE v. 2.033, and GapFiller v 1.134 followed by annotation using Prokka v. 1.5–135. These genomes are available on the European Nucleotide Archive under the accession numbers ERS2102305 (*Hp* GFP) and ERS2102306 (*Hp* tdT).

### Genome analysis

SNPs were detected by first mapping the reads with SMALT v. 0.7.4 against the WT genome and subsequently generating a compressed variant call format file using a combination of samtools v. 0.1.19 and bcftools v. 0.1.19. Roary [69] was used to carry out the pan-genome analysis, and results were visualized in Artemis [70]. Genome similarity was calculated using the average nucleotide identity.

### Animal work ethics statement

All animal experiments were performed in accordance with NIH guidelines, with approval by the Stanford University Administrative Panel on Laboratory Animal Care (APLAC) and overseen by the Institutional Animal Care and Use Committee (IACUC) under Protocol ID 9677. Mouse experiments performed at UC Davis were approved by the UC Davis IACUC under Protocol ID 18849. Animals were housed in a research animal facility that is accredited by the Association of Assessment and Accreditation of Laboratory Animal Care (AAALAC) International.

### Mouse strains and husbandry

For a majority of mouse experiments performed, WT C57BL/6J female mice (6–8 weeks old; Stock #000664) were purchased from Jackson Laboratories (Bar Harbor, ME). Mice were given at least 5–7 days to acclimate to the Stanford Animal Biohazard Research Facility prior to infection with *H*. *pylori*.

For neonatal mouse infections, WT C57BL/6 male mice were crossed with C57BL/6J female mice purchased from Jackson Laboratories (Stock #000664) to obtain pups for experiments. Both male and female pups were infected with *H*. *pylori* when they were 1 week old.

For experiments involving mice lacking functional T cells, TCRβ/δ^−/−^ mice in the C57BL/6J background were purchased from Jackson Laboratories (Stock #002122) along with WT C57BL/6J mice (Stock #000664) used as controls. Mouse colonies of both strains were maintained at UC Davis. Both male and female mice from the colonies (6–8 weeks old) were used for experiments.

Mice were housed under specific pathogen-free conditions in filter-top cages that were changed once a week by veterinary or research personnel. Sterile water and food (2018 Teklad Global 18% Protein Rodent Diet; Envigo, Indianapolis, IN) were provided as needed.

### Animal experiments

Animals (6–8-week-old females) were orally infected by feeding them a 5-μL suspension containing 10^8^ CFU of *H*. *pylori* grown in BB10, as previously described [11]. For 1:1 competition infections, mice were infected with an equal mixture of *Hp* GFP and *Hp* tdT, totaling 10^8^ CFU. For sequential infections, mice were infected with 10^8^ CFU of the initial strain for 1 week and then infected with 10^8^ CFU of the challenge strain for 1 week (total, 2-week infection). For sequential infections with antibiotic treatment, animals were infected with 10^8^ CFU of *Hp* GFP for 1 week and then treated with amoxicillin at 16 mg/kg/day via drinking water for 3 days. Afterwards, amoxicillin was removed from the drinking water overnight, and the animals were inoculated with 10^8^ CFU of *Hp* tdT the next morning.

For experiments involving neonatal mice, 1-week-old mice were orally infected by feeding them a 5-μL suspension containing 10^8^ CFU of *H*. *pylori* grown in BB10 (one dose per day for 2 consecutive days). Infected pups are caged with their mothers until weaning at 3–4 weeks of age.

For experiments involving mice lacking functional T cells, TCRβ/δ^−/−^ or WT mice (6–8-week-old males and females) were challenged with 10^8^ CFU of *H*. *pylori* suspended in 0.25 mL of Brucella broth administered by oral gavage.

Stomachs were harvested from infected animals at different time points post-infection. First, the forestomach was removed and discarded. Subsequently, the stomach was opened via the lesser curvature, laid flat on a piece of filter paper, and the luminal contents were removed. The stomach was then divided into two halves that spanned both the corpus and antrum. One half was weighed and mechanically homogenized in Brucella broth to enumerate CFU/g of stomach; the other half was fixed in 2% paraformaldehyde in 100 mM phosphate buffer (pH 7.4) at room temperature for 1 hour. Infections were conducted with 3–6 animals per group and were repeated two to three times to ensure reproducibility.

### Confocal microscopy of longitudinal sections

Tissue samples from infected murine stomachs were processed for confocal immunofluorescence microscopy as previously described [11,13,19]. Briefly, stomach tissues fixed in 2% paraformaldehyde in 100 mM phosphate buffer (pH 7.4) were embedded in 4% agarose, and 150 μm longitudinal sections were generated using a Vibratome (Leica, Solms, Germany). Tissue sections were permeabilized and blocked in phosphate-buffered saline (PBS) containing 3% bovine serum albumin, 1% saponin, and 1% Triton X-100 prior to staining. Samples were imaged with a Zeiss LSM 700 confocal microscope (Carl Zeiss, Oberkochen, Germany). Quantification of the number of *H*. *pylori* per gastric gland in *Hp* GFP or *Hp* tdT single-infected animals was performed using Volocity v5.5 Image Analysis software (Perkin Elmer, Santa Clara, CA), as previously described [11,13,19]. Briefly, bacterial microcolony signals are identified by fluorescence intensity and size on Volocity. Subsequently, the identified voxels are measured, and the number of bacteria per gland is calculated by dividing the measured values by the average voxel volume of one bacterium.

### Mapping location of gland-associated bacteria

To map the distribution of gland-associated *H*. *pylori* within infected stomachs, 6-μm-thick composite images of longitudinal sections through the antrum and transition zone were generated and tiled using Adobe Photoshop. The composite images were thresholded so background signal would not be measured as bacteria. The position of each gland along the x-axis and the height of each gland were determined using ImageJ software and subsequently converted to a μm scale. To determine the number of *Hp* GFP and *Hp* tdT per gland, we measured individually the integrated density of the green and red signals present in each gland within the section. The integrated density was then divided by the average integrated density per bacterium to obtain the number of bacteria per gland. When plotting the gland distribution data, we set the junction between the antrum and transition zone as x = 0 (anything that is negative distance is antrum, and anything that is positive distance is transition zone).

### Passive CLARITY of intact stomach tissue

Intact stomach tissues from infected mice were processed for confocal immunofluorescence microscopy utilizing PACT, as previously described [20,21]. Briefly, stomach tissues fixed in 2% paraformaldehyde in 100 mM phosphate buffer (pH 7.4) were incubated at 4 °C for 1–2 days in the hydrogel monomer solution A4P0 (4% acrylamide in PBS) supplemented with 0.25% thermoinitiator 2,2′-Azobis[2-(2-imidazolin-2-yl)propane]dihydrochloride (VA-044; Oxchem, Wood Dale, IL, Catalog #AX8064242). Subsequently, A4P0-infused samples were degassed with nitrogen for 5 minutes and then incubated for 3–4 hours in a 37 °C water bath to initiate tissue-hydrogel hybridization. Excess hydrogel was removed from the samples via several brief PBS washes before transferring the samples into 50-mL conical tubes containing 8% SDS in PBS (pH 7.4). The samples were incubated in 8% SDS/PBS at 37 °C, shaking gently, for 1–2 days. Afterwards, the cleared tissue samples were washed in PBS with 4–5 buffer changes over the course of a day before being transferred to staining solutions containing appropriate primary antibodies for 7 days. Unbound antibodies were removed via PBS washes as before, and then samples were incubated with fluorophore-conjugated secondary antibodies for another 7 days. Finally, stained samples are washed in PBS throughout the course of a day, as before, and then incubated in refractive index matching solution (RIMS) mounting medium overnight. To generate RIMS with a refractive index of 1.47, which is used for all samples in this paper, 10 g of Histodenz (Sigma-Aldrich) were dissolved in 7.5 mL of 0.02 M phosphate buffer. Staining buffer is composed of PBS containing 3% bovine serum albumin, 1% saponin, and 1% Triton X-100. Primary and secondary antibody solutions were replaced at least once during the staining process. All staining procedures were done at room temperature with gentle rotation.

### Antibodies and tissue-labeling reagents

Goat anti-GFP (R&D Systems, Minneapolis, MN, Catalog #AF4240; RRID: AB_884445), rabbit anti-RFP (Rockland, Limerick, PA, Catalog #600-401-379; RRID: AB_2209751), and appropriate secondary antibodies (Alexa Fluor 488– or Alexa Fluor 594–conjugated; Invitrogen) were used to visualize fluorescent *Hp* GFP and *Hp* tdT, respectively, in gastric tissue. A custom-generated rabbit anti–*H*. *pylori* PMSS1 antibody and appropriate secondary antibodies were used to visualize PMSS1 WT bacteria [11]. DAPI (4’,6-Diamidino-2-phenylindole) and Alexa Fluor 594 or 660 phalloidin (Invitrogen) were used for visualization of nuclei and F-actin, respectively.

### Simulation of gland colonization and spread

Wolfram Mathematica 11.3 was used to simulate the spread of two strains of *H*. *pylori* across gastric glands that were distributed in a grid, together forming a tube with a length of 200 glands and a circumference of 100 glands. We therefore assumed a total of 20,000 glands available for bacterial colonization, which roughly corresponds to the number of glands in a mouse’s gastric antrum and transition zone. In the co-infection simulations, both strains (*Hp* GFP and *Hp* tdT) were introduced at the same time. Ten glands were randomly selected for initial colonization via oral infection of *Hp* GFP. Similarly, ten glands were selected for colonization by *Hp* tdT. At each time step *t* (hours), each gland adjacent to each of those occupied by either *Hp* GFP or *Hp* tdT was colonized by that strain with a probability of Exp[m*t/24], where *m* is the rate of increase in the total number of bacteria during the first 5 days after introduction. To estimate *m*, we assumed an exponential growth, in which the number of colonized glands was *P*_0_*e^*mt*^. Using CFU data from the single-strain introduction experiment (S3A Fig), we estimated *m* = 0.709 (*r*^2^ = 0.997). Every 8 hours, the bacteria in colonized glands will move to adjacent glands with a fixed probability. If both *Hp* GFP and *Hp* tdT were simultaneously selected for colonization of a given unoccupied gland, we assumed that the gland would be colonized by both. We also assumed that once *Hp* GFP occupies an empty gland, *Hp* tdT can still colonize that gland if it arrives within 168 hours (7 days) after *Hp* GFP arrival (and vice versa), prior to saturation of the gland space. The gland would then become co-occupied. But if *Hp* tdT did not arrive within 168 hours (7 days), the gland would remain *Hp* GFP dominated and become resistant to colonization by *Hp* tdT (and vice versa). From the glands that are co-occupied, each of *Hp* GFP and *Hp* tdT spread to half of the adjacent glands selected for colonization. We ran the simulation for 28 days.

We tried three different simulation scenarios. The first scenario had both of the assumptions that we built in to reproduce the experimental results. The other scenarios lacked one of these assumptions (Fig 3E and S4 Fig). Specifically, we assumed the following in Scenario 1: (a) the bacteria can only spread from occupied glands to unoccupied glands that are directly adjacent (adjacent spread), and (b) once a gland is colonized to saturation, that population remains in that gland, and no other bacteria can establish (gland resistance). In Scenario 2, we relaxed the gland resistance parameter by allowing each strain to colonize adjacent glands, regardless of how long the glands had been occupied by the other strain. We assumed that these glands would become co-occupied by both strains over time. In Scenario 3, we relaxed the adjacent spread parameter by implementing adjacent colonization for 90% of the colonization events and, for the other 10%, randomly choosing a gland from the entire stomach and colonizing that gland, if available (distant spread).

### Statistical analysis

GraphPad Prism 7 was used to generate all figures and perform statistical tests for *H*. *pylori* mouse infections. For comparison of CFU burdens, statistical significance was assessed via a Mann–Whitney test (for single infections) or a Wilcoxon signed-rank test (for co-infections). For comparison of numbers of bacteria per gland, a Mann–Whitney test was used. Center values are medians unless otherwise noted. NS indicates no statistical significance, *p < 0.05, **p < 0.01, ***p < 0.001, and ****p < 0.0001.

## Supporting information

S1 Fig Generation of isogenic and equally fit fluorescent *H*. *pylori* strains. (A) To generate isogenic fluorescent *H*. *pylori* strains in the PMSS1 background (*Hp* GFP or *Hp* tdT), the nonessential gene *rdxA* was replaced via homologous recombination by a construct containing the *aphA* gene (conferring kanamycin resistance, kan^R^), the *ureA* promoter, and either the *gfp* or *tdTomato* gene (FP). Expression of the fluorophore genes was driven by the *ureA* promoter. *rdxA* is a nonessential locus commonly used for complementation of *H*. *pylori* knockout mutants [11]. (B) A 1:1 mixture of *Hp* GFP (green) and *Hp* tdT (red) strains. Scale bar, 10 μm. (C) In vitro growth curves showing OD_600_ readings of *Hp* GFP and *Hp* tdT strains grown individually in broth for 28 hours. Both strains double about every 4 hours, and no differences were observed. Data represent biological triplicates for each strain. Error bars, standard deviation. Relevant data values are included in S1 Data. (D) Bacterial attachment assay. AGS cells were infected with either *Hp* GFP or *Hp* tdT, and the number of attached bacteria was enumerated one minute after initial attachment. Data represent biological triplicates for each strain. Bars, median; error bars, interquartile range. Statistics: *p*-value obtained using a Mann–Whitney test. Relevant data values are included in S1 Data. (E) CagA delivery assay. AGS cells were cocultured with *Hp* GFP, *Hp* tdT, *Hp* GFP Δ*cagE*, or *Hp* tdT Δ*cagE* for 24 hours. Three-dimensional confocal immunofluorescence images show elongation of cells infected with fluorescent WT strains (a phenotype consistent with CagA injection), but not with fluorescent Δ*cagE* strains. Nuclei (blue), F-actin (white); scale bar, 30 μm. (F) Fluorescent bacteria are mixed in the surface mucus. Three-dimensional confocal image of a patch of preserved mucus from a 2-week co-infected stomach. Scale bar, 10 μm. CFU, colony-forming unit; FP, fluorescent protein; GFP, green fluorescent protein; NS, no significance; OD, optical density; tdT, tdTomato; WT, wild-type.
(TIF)

S2 Fig Organization of *H*. *pylori* gland populations as patches occur in multiple co-infected animals. Mapping of location and number of gland-associated bacteria across longitudinal stomach sections from additional animals co-infected with *Hp* GFP and *Hp* tdT at 2 weeks post-infection. Each plot represents a single section from one individual mouse. The three plots labeled with an asterisk (*) are three sections at least 300 μm apart from each other that were taken from the same co-infected animal. Gland height (black line) and bacteria per gland (green and red bars) are mapped according to their location within the section. x = 0 marks the junction between the antrum and transition zone. Total CFU/g recovered from each mouse indicated. CFU, colony-forming unit; GFP, green fluorescent protein; tdT, tdTomato.
(TIF)

S3 Fig Gland population islands originate from a small number of founder bacteria. (A) *H*. *pylori* experiences a huge bottleneck when establishing initial colonization. Total CFU recovered from a whole stomach at various time points post-infection with *Hp* GFP (3–5 mice per time point). All mice were infected as adults. “Inoc” represents the 10^8^ CFU inoculum given per animal. Black bars, geometric mean. Data represent two independent experiments. Relevant data values are included in S1 Data. (B) Mapping of location and number of gland-associated bacteria across longitudinal stomach sections from animals infected with *Hp* GFP for 3, 5, or 7 days. Each plot represents a single section from one individual mouse from each time point. Gland height (black line) and bacteria per gland (green bars) are mapped according to their location within the section. x = 0 marks the junction between the antrum and transition zone. Total CFU/g recovered is depicted for each animal analyzed. (C) Total CFU/g recovered from mice infected with a 1:1 mixture of *Hp* GFP and *Hp* tdT for 5 days (six mice). Black dashed lines connect the *Hp* GFP and *Hp* tdT counts recovered from the same individual mouse. Data represent two independent experiments. Gray dotted line, limit of detection. Statistics: *p*-value obtained using Wilcoxon signed-rank test. Relevant data values are included in S1 Data. (D) Representative PACT images of clonal islands found in 5-day co-infected mice. Nuclei (blue), GFP (green), tdTomato (red); scale bar, 160 μm. CFU, colony-forming unit; GFP, green fluorescent protein; NS, no significance; PACT, passive CLARITY technique; tdT, tdTomato.
(TIF)

S4 Fig Computer simulations that assume adjacent spread and gland resistance recapitulate observations in murine co-infections. Simulation of *H*. *pylori* gland colonization and spread, in which 10 randomly selected glands were colonized by *Hp* GFP and another set of 10 by *Hp* tdT in a field of 20,000 available glands, and bacteria were allowed to spread over time. Three different scenarios including or excluding the assumptions of adjacent spread and gland resistance were implemented. In our simplified model, only three gland types can arise when colonized: green, *Hp* GFP–occupied glands; red, *Hp* tdT–occupied glands; yellow, 50:50 co-occupied glands. Shown here are the percentages of total colonized glands for each gland type for each scenario at 14 or 28 days post-infection. Data represent 10 replicated simulation runs per scenario (each dot represents a value from each replicate). Bars, arithmetic mean. Results from actual murine co-infections shown for comparison, in which most glands are composed of a single color and 50:50 co-occupied glands are rare, and this distribution persists over time. Relevant data values are included in S1 Data. GFP, green fluorescent protein; tdT, tdTomato.
(TIF)

S5 Fig Mice infected as neonates maintain a higher density of gland-associated bacteria during chronic infection. (A) Total CFU/g recovered from mice infected as 1-week-old neonates or 6-week-old adults at 1 month post-infection (8–9 animals per group). These are the same 1-month co-infected animals from Fig 3. Gray dotted line, limit of detection; red bars, median. Data represent two independent experiments. Relevant data values are included in S1 Data. (B) Bacteria per gland in mice infected as adults or neonates. Red bars, median. A total of 150–200 glands were analyzed per mouse (three mice per group). Relevant data values are included in S1 Data. (C) Images of gland-associated bacteria from mice colonized as adults or neonates. Scale bar, 80 μm. Statistics: *p*-value obtained using a Mann–Whitney test (panels A, B). **p < 0.01, ****p < 0.0001. CFU, colony-forming unit.
(TIF)

S1 Table Differences between *Hp* GFP and *Hp* tdT genomes. (PDF)

S2 Table Primers used to generate fluorophore-expression constructs. (PDF)

S1 Movie Mapping *H*. *pylori* biogeography in longitudinal gastric sections. (MOV)

S2 Movie High spatial accuracy and resolution analysis of *H*. *pylori* gastric colonization with passive CLARITY technique (PACT) and 3D confocal microscopy. (MOV)

S1 Data (XLSX)

## Abbreviations

3D: three-dimensional
AI-2: autoinducer-2
CFU: colony-forming unit
GFP: green fluorescent protein
*Hp* GFP: *H*. *pylori* GFP-expressing strain
*Hp* tdT: *H*. *pylori* tdTomato-expressing strain
IFNγ: interferon gamma
PACT: passive CLARITY technique
ROS: reactive oxygen species
SDS: sodium dodecyl sulfate
SNP: single nucleotide polymorphism
TCR: T-cell receptor
tdT: tdTomato
Th1: T-helper 1
WT: wild-type
