# Supplementary figures and images for: High-resolution mapping reveals that microniches in the gastric glands control Helicobacter pylori colonization of the stomach
Source: PLoS Biol. 2019 May 2;17(5):e3000231. doi: 10.1371/journal.pbio.3000231 (PMC6497225; doi:10.1371/journal.pbio.3000231)

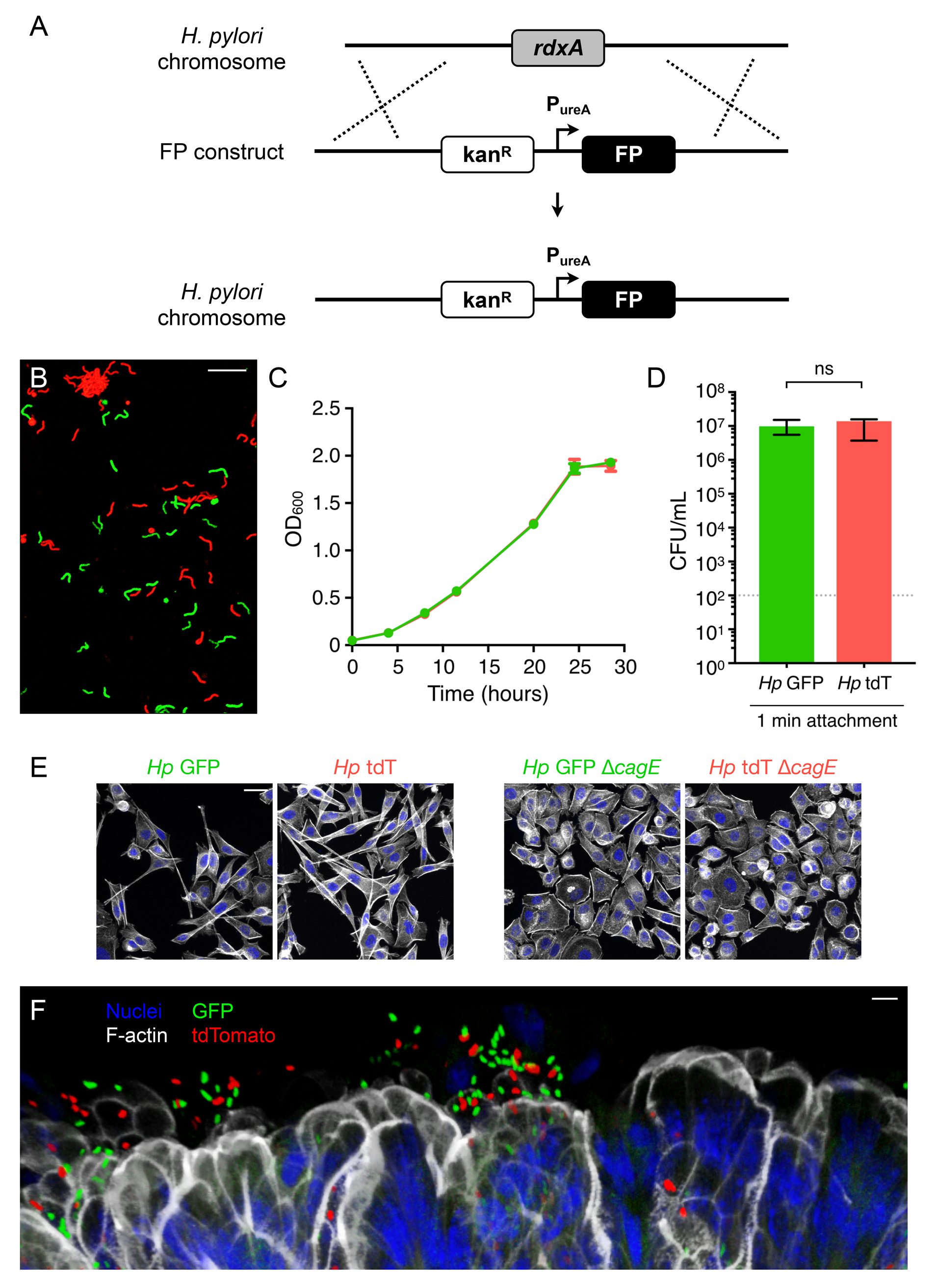

Supplement: S1 Fig — (A) To generate isogenic fluorescent H. pylori strains in the PMSS1 background (Hp GFP or Hp tdT), the nonessential gene rdxA was replaced via homologous recombination by a construct containing the aphA gene (conferring kanamycin resistance, kanR), the ureA promoter, and either the gfp or tdTomato gene (FP). Expression of the fluorophore genes was driven by the ureA promoter. rdxA is a nonessential locus commonly used for complementation of H. pylori knockout mutants [11]. (B) A 1:1 mixture of Hp GFP (green) and Hp tdT (red) strains. Scale bar, 10 μm. (C) In vitro growth curves showing OD600 readings of Hp GFP and Hp tdT strains grown individually in broth for 28 hours. Both strains double about every 4 hours, and no differences were observed. Data represent biological triplicates for each strain. Error bars, standard deviation. Relevant data values are included in S1 Data. (D) Bacterial attachment assay. AGS cells were infected with either Hp GFP or Hp tdT, and the number of attached bacteria was enumerated one minute after initial attachment. Data represent biological triplicates for each strain. Bars, median; error bars, interquartile range. Statistics: p-value obtained using a Mann–Whitney test. Relevant data values are included in S1 Data. (E) CagA delivery assay. AGS cells were cocultured with Hp GFP, Hp tdT, Hp GFP ΔcagE, or Hp tdT ΔcagE for 24 hours. Three-dimensional confocal immunofluorescence images show elongation of cells infected with fluorescent WT strains (a phenotype consistent with CagA injection), but not with fluorescent ΔcagE strains. Nuclei (blue), F-actin (white); scale bar, 30 μm. (F) Fluorescent bacteria are mixed in the surface mucus. Three-dimensional confocal image of a patch of preserved mucus from a 2-week co-infected stomach. Scale bar, 10 μm. CFU, colony-forming unit; FP, fluorescent protein; GFP, green fluorescent protein; NS, no significance; OD, optical density; tdT, tdTomato; WT, wild-type. (TIF) [file pbio.3000231.s001.tif]

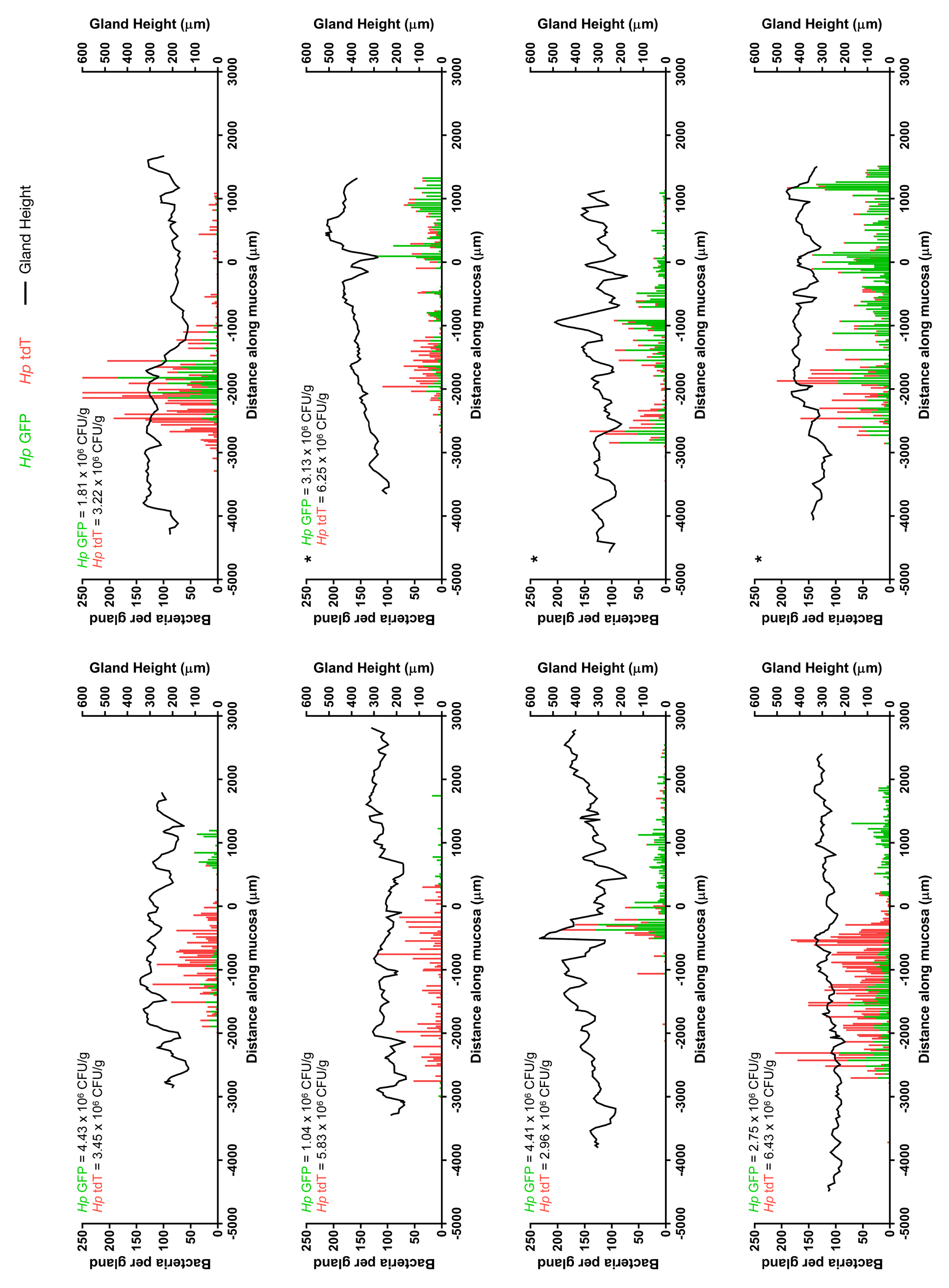

Supplement: S2 Fig — Mapping of location and number of gland-associated bacteria across longitudinal stomach sections from additional animals co-infected with Hp GFP and Hp tdT at 2 weeks post-infection. Each plot represents a single section from one individual mouse. The three plots labeled with an asterisk (*) are three sections at least 300 μm apart from each other that were taken from the same co-infected animal. Gland height (black line) and bacteria per gland (green and red bars) are mapped according to their location within the section. x = 0 marks the junction between the antrum and transition zone. Total CFU/g recovered from each mouse indicated. CFU, colony-forming unit; GFP, green fluorescent protein; tdT, tdTomato. (TIF) [file pbio.3000231.s002.tif]

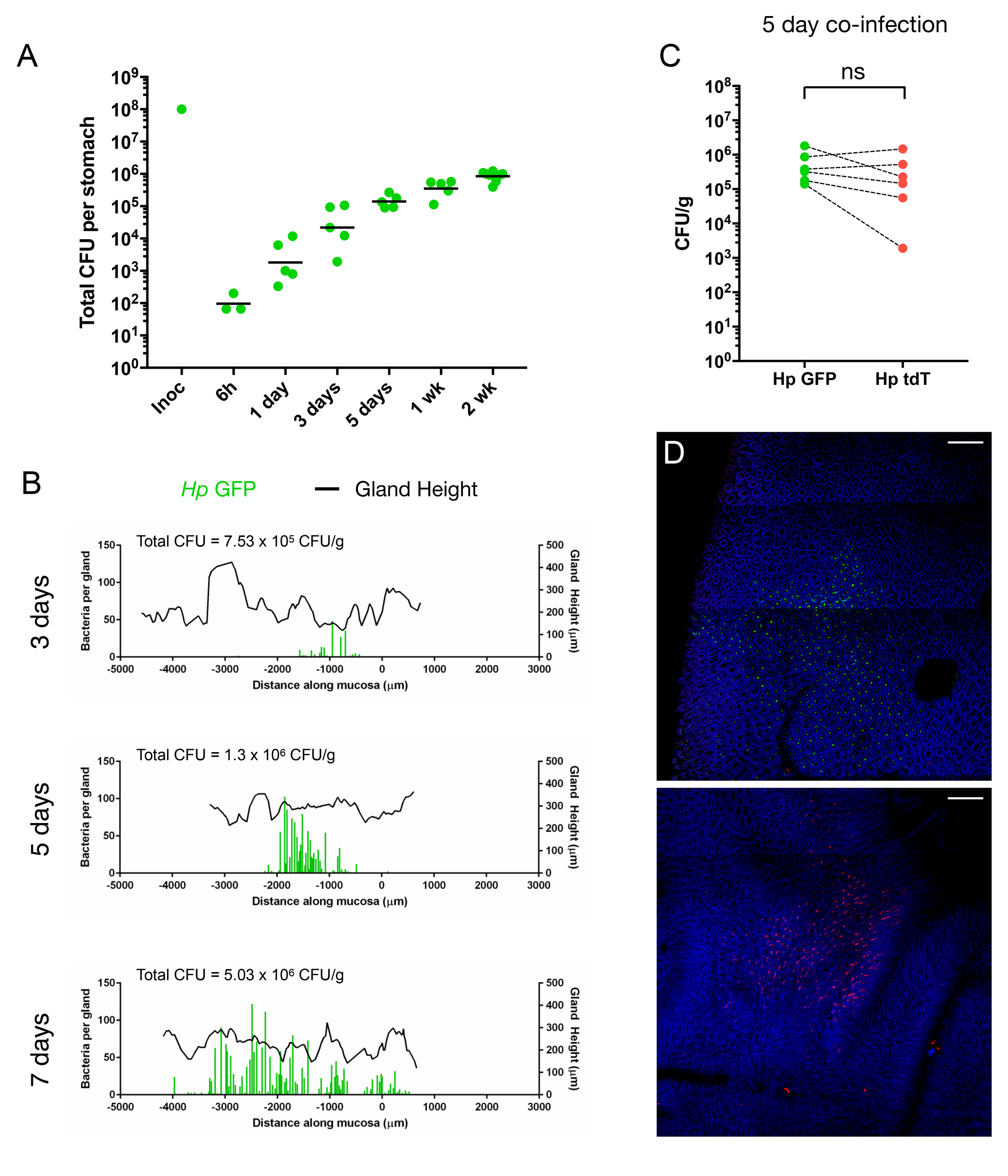

Supplement: S3 Fig — (A) H. pylori experiences a huge bottleneck when establishing initial colonization. Total CFU recovered from a whole stomach at various time points post-infection with Hp GFP (3–5 mice per time point). All mice were infected as adults. “Inoc” represents the 108 CFU inoculum given per animal. Black bars, geometric mean. Data represent two independent experiments. Relevant data values are included in S1 Data. (B) Mapping of location and number of gland-associated bacteria across longitudinal stomach sections from animals infected with Hp GFP for 3, 5, or 7 days. Each plot represents a single section from one individual mouse from each time point. Gland height (black line) and bacteria per gland (green bars) are mapped according to their location within the section. x = 0 marks the junction between the antrum and transition zone. Total CFU/g recovered is depicted for each animal analyzed. (C) Total CFU/g recovered from mice infected with a 1:1 mixture of Hp GFP and Hp tdT for 5 days (six mice). Black dashed lines connect the Hp GFP and Hp tdT counts recovered from the same individual mouse. Data represent two independent experiments. Gray dotted line, limit of detection. Statistics: p-value obtained using Wilcoxon signed-rank test. Relevant data values are included in S1 Data. (D) Representative PACT images of clonal islands found in 5-day co-infected mice. Nuclei (blue), GFP (green), tdTomato (red); scale bar, 160 μm. CFU, colony-forming unit; GFP, green fluorescent protein; NS, no significance; PACT, passive CLARITY technique; tdT, tdTomato. (TIF) [file pbio.3000231.s003.tif]

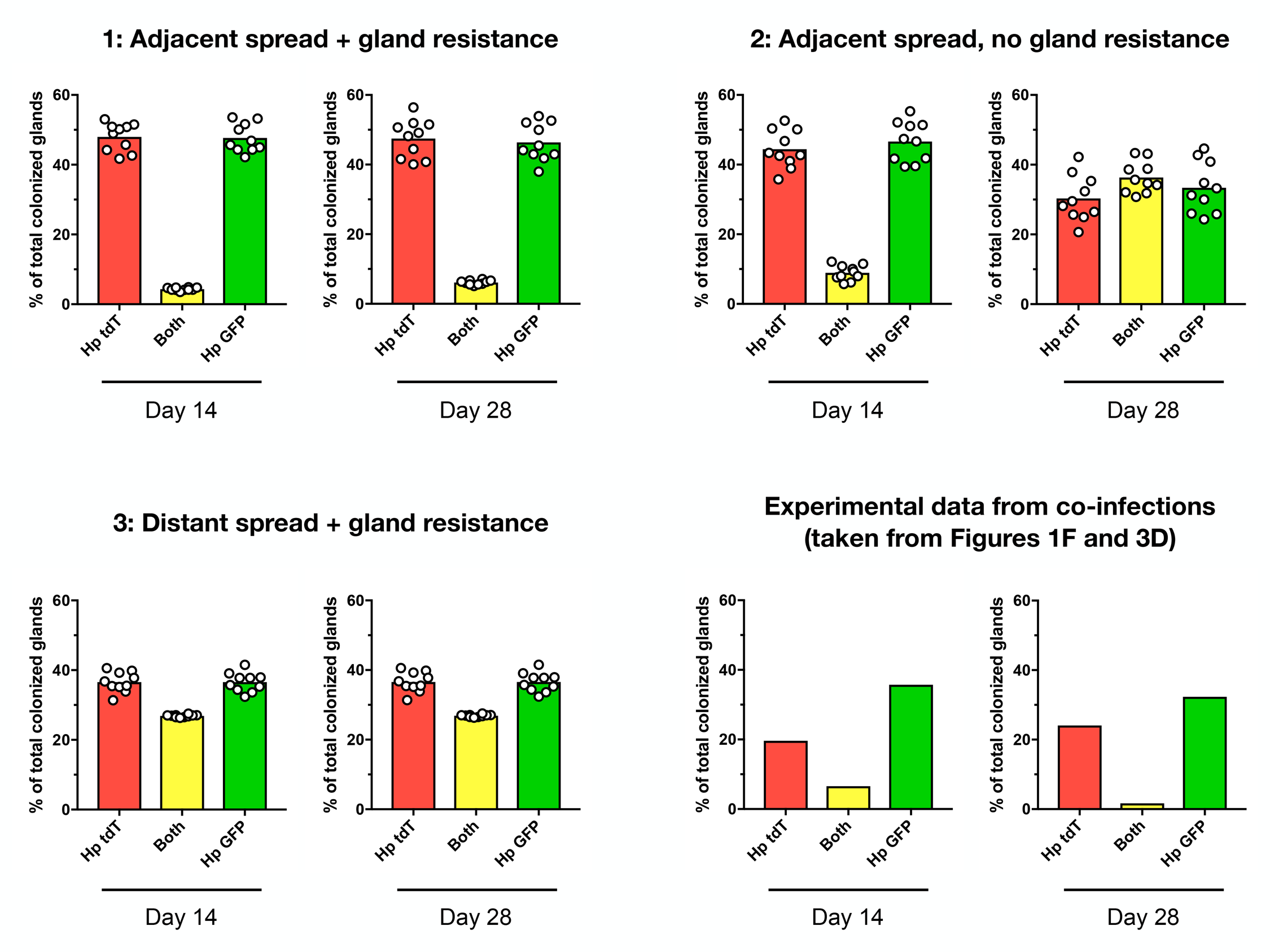

Supplement: S4 Fig — Simulation of H. pylori gland colonization and spread, in which 10 randomly selected glands were colonized by Hp GFP and another set of 10 by Hp tdT in a field of 20,000 available glands, and bacteria were allowed to spread over time. Three different scenarios including or excluding the assumptions of adjacent spread and gland resistance were implemented. In our simplified model, only three gland types can arise when colonized: green, Hp GFP–occupied glands; red, Hp tdT–occupied glands; yellow, 50:50 co-occupied glands. Shown here are the percentages of total colonized glands for each gland type for each scenario at 14 or 28 days post-infection. Data represent 10 replicated simulation runs per scenario (each dot represents a value from each replicate). Bars, arithmetic mean. Results from actual murine co-infections shown for comparison, in which most glands are composed of a single color and 50:50 co-occupied glands are rare, and this distribution persists over time. Relevant data values are included in S1 Data. GFP, green fluorescent protein; tdT, tdTomato. (TIF) [file pbio.3000231.s004.tif]

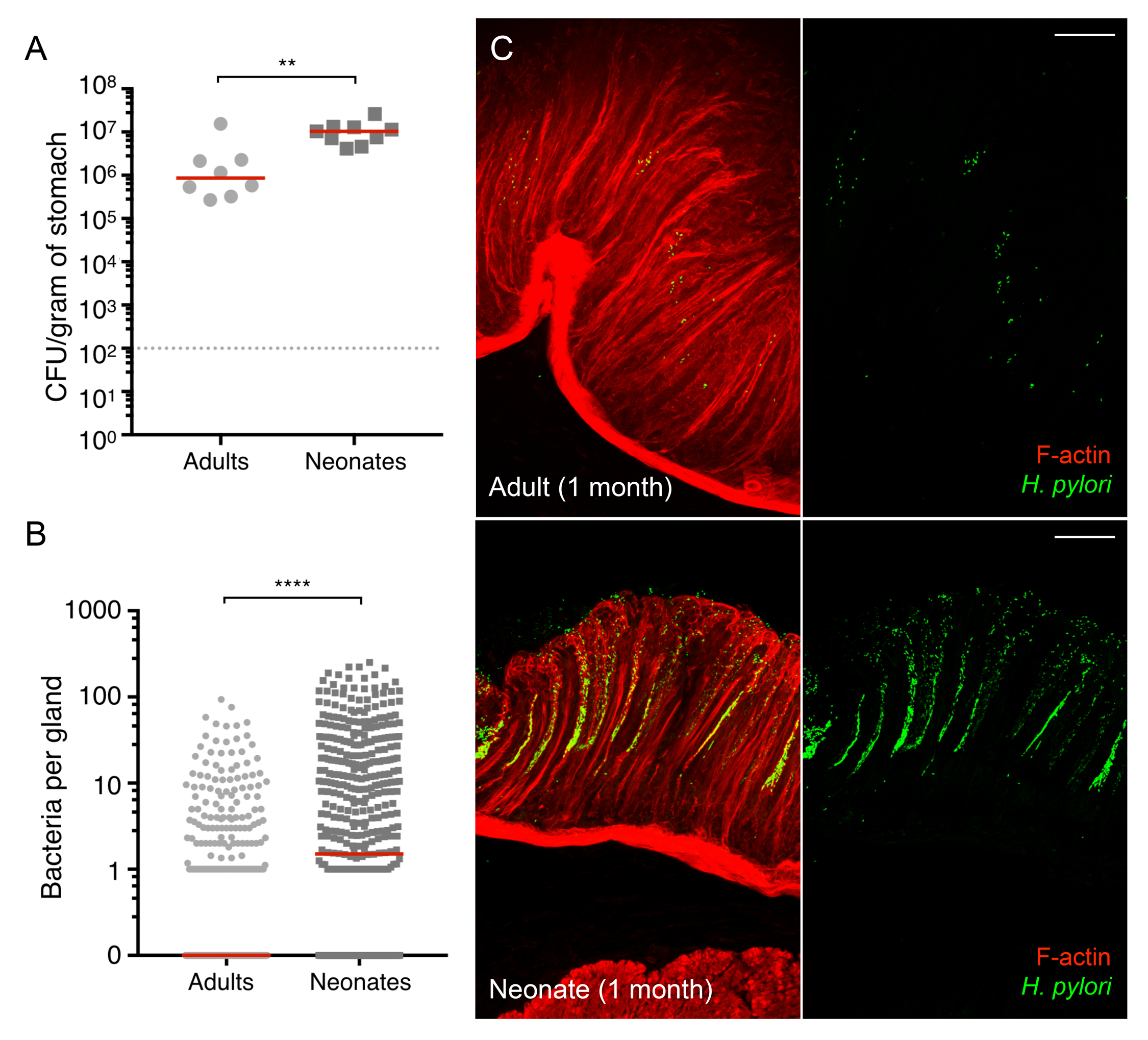

Supplement: S5 Fig — (A) Total CFU/g recovered from mice infected as 1-week-old neonates or 6-week-old adults at 1 month post-infection (8–9 animals per group). These are the same 1-month co-infected animals from Fig 3. Gray dotted line, limit of detection; red bars, median. Data represent two independent experiments. Relevant data values are included in S1 Data. (B) Bacteria per gland in mice infected as adults or neonates. Red bars, median. A total of 150–200 glands were analyzed per mouse (three mice per group). Relevant data values are included in S1 Data. (C) Images of gland-associated bacteria from mice colonized as adults or neonates. Scale bar, 80 μm. Statistics: p-value obtained using a Mann–Whitney test (panels A, B). **p < 0.01, ****p < 0.0001. CFU, colony-forming unit. (TIF) [file pbio.3000231.s005.tif]
