# Supplementary material for: High-resolution mapping reveals that microniches in the gastric glands control Helicobacter pylori colonization of the stomach
Source: PLoS Biol. 2019 May 2;17(5):e3000231. doi: 10.1371/journal.pbio.3000231 (PMC6497225; doi:10.1371/journal.pbio.3000231)
Supplement: S1 Table — (PDF) [file pbio.3000231.s006.pdf]

Gene	Description	Difference
rpoA	DNA-directed RNA polymerase subunit alpha	6 SNPs, no amino acid differences
cstA	carbon starvation protein A	2 SNPs, no amino acid differences
vacA_1	vacuolating cytotoxin (VacA) family protein	1 SNP, no amino acid differences
accD	acetyl-CoA carboxylase subunit beta	1 SNP, no amino acid differences
Lgt	prolipoprotein diacylglyceryl transferase	5 SNPs, resulting in 1 amino acid difference
Alpha 1,3-fucosyltransferase	Alpha 1,3-fucosyltransferase	6 SNPs, resulting in 4 amino acid differences
dcuA	anaerobic C4-dicarboxylate transporter	1 SNP, no amino acid differences

S1 Table. Differences between *Hp* GFP and *Hp* tdT genomes.
