# Supplementary material for: High-resolution mapping reveals that microniches in the gastric glands control Helicobacter pylori colonization of the stomach
Source: PLoS Biol. 2019 May 2;17(5):e3000231. doi: 10.1371/journal.pbio.3000231 (PMC6497225; doi:10.1371/journal.pbio.3000231)
Supplement: S2 Table — (PDF) [file pbio.3000231.s007.pdf]

GFP **expression construct**	
**Primer Name**	**Sequence (5' to 3')**
rdxA 5' flank forward	ggcggagcttggcattg
rdxA 5' flank reverse	atggttcgctgggtttatctgattttcctttattttttagaatg
rdxA 3' flank forward	ggatgaattgttttagtaccttaaacaaaatcaaaaactttttaac
rdxA 3' flank reverse	ccctaaacatgctcgctag
aphA + *ureA* prom + GFP forward	agacatctaaatctaggtactaa
aphA + *ureA* prom + GFP reverse	ttatttgtatagttcatccatgc
tdTomato **expression construct**	
**Primer Name**	**Sequence (5' to 3')**
rdxA 5' flank forward	ggcggagcttggcattg
rdxA 5' flank reverse	atggttcgctgggtttatctgattttcctttattttttagaatg
rdxA 3' flank forward	ggatgaattgttttagtaccttaaacaaaatcaaaaactttttaac
rdxA 3' flank reverse	ccctaaacatgctcgctag
aphA + *ureA* prom forward	agacatctaaatctaggtactaa
aphA + *ureA* prom reverse	cttcgccttttgacaccatctcattctcctattcttaaagtg
tdTomato forward	atggtgtcaaaaggcgaag
tdTomato reverse	ttatttgtataattcatccatacc

S2 Table. Primers used to generate fluorophore-expression constructs.
